# Insights into neuroscience from the representative birth cohort samples of a multidisciplinary longitudinal study

**DOI:** 10.1017/neu.2026.10072

**Published:** 2026-04-07

**Authors:** Jaanus Harro, Diva Eensoo, Evelyn Kiive, Inga Villa, Triin Kurrikoff, Jarek Mäestu, Margus Kanarik, Karita Laugus, Katre Sakala, Toomas Veidebaum

**Affiliations:** 1 Division of Neuropsychopharmacology, Institute of Chemistry, University of Tartu, Estonia; 2 Department of Chronic Diseases, National Institute for Health Development, Estonia; 3 Division of Special Education, Department of Education, University of Tartu, Estonia; 4 Department of Family Medicine and Public Health, University of Tartu, Estonia; 5 Department of Exercise Biology, Institute of Sport Sciences and Physiotherapy, University of Tartu, Estonia

**Keywords:** longitudinal studies, behavioural medicine, gene × environment interaction, birth cohort, gender

## Abstract

Longitudinal studies on population representative samples offer unique insights. The Estonian Children Personality Behaviour and Health Study (ECPBHS; EstChild) was launched in 1998 on two birth cohort samples at age 9 or 15 with an exceptional participation rate, has been monitored at ages 15, 18, 25 and 33, and also recruited parents of the target subjects. This multidisciplinary investigation has been focused on behavioural neuroscience, illuminating findings on what could be discerned from biomarkers, candidate genes, gene × environment interactions, and epigenetic markers in representative samples, and in birth cohorts living through societal transformation. ECPBHS analysed how biomarkers and lifestyle are associated with real-life behaviours and developmental trajectories, phenotypes such as neuroticism, bulimia, aggressiveness or attention deficit, and outcomes from incidence of psychiatric disorders to the obtaining of university education. Novel evidence has been observed on clustering of fears and the inner structure of impulsivity and reward sensitivity, together with clues how these may have co-emerged with metabolic types. New insights have been provided to understand the classic biomarkers, cholesterol and platelet monoamine oxidase activity, as well as several functional gene variants. Hypotheses how to synthetise molecular genetics and sociology, how sex or gender matters in the light of gene × environment interactions and how family and parental roles shape the behaviour of offspring have been put forward. The ECPBHS has offered clues on why in biological psychiatry many replication attempts are predestined to fail, and how to learn from such failures.

## Graphical abstract

**Figure:**
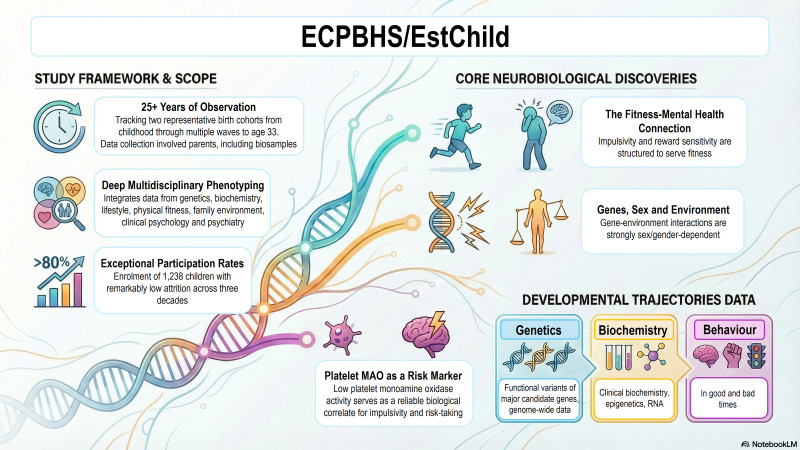

Summations

- Longitudinal design enables the study of developmental stages of behaviour.
- Deeply phenotyped highly representative birth cohort samples help to detect reliable biomarkers and understand their relationships with adaptive strategies.
- Gene × environment interactions differ gender-wise.

Perspectives

- Longitudinal design is setting limits to sample size, and hence to more granular investigation of distinct developmental curves. Pooling data of similar studies and replication testing are important for validation of the findings.
- Environmental variability is impeding accurate reproducibility, unless the salient features in ‘enviromics’ are identified.
- Adaptive strategies and gender differences are better interpreted in the broader socio-cultural context.

## Introduction

Longitudinal studies with deep phenotyping have provided valuable insights into human development and etiopathogenesis of disease, perhaps most importantly for cardiovascular health (Dawber *et al*., 1963; Caruana *et al*., 2015; Andersson *et al*., 2019). While mental and physical health are often treated as separate categories, the impact of mental health on systemic health and vice versa is difficult to underestimate (Prince *et al*., 2007). Because of common risk factors, the impacts of health behaviour and shared diathesis mechanisms are indeed inseparable. More recently, several longitudinal studies on patient as well as community cohorts have provided important data on depression and other mental health conditions, their co-morbidities and association with physiological and behavioural risk factors (Luppino *et al*., 2010; McKay *et al*., 2021; Palmese *et al*., 2025). Herewith we review the procedure and neuropsychiatrically relevant findings of the Estonian Children Personality Behaviour and Health Study (ECPBHS; EstChild), an investigation of highly representative birth cohort samples conducted since 1998. While some results of this study have been reported since 2001 (the list of ECPBHS journal articles and book chapters is available at https://www.tai.ee/et/teadustoo/eliktu-artiklid), the breadth of this study has not yet been comprehensively described. The ECPBHS has collected a highly multidisciplinary dataset, but it is the neuropsychiatric dimension that has received most attention, unveiling several provocative findings.

## How the ECPBHS has been conducted: methods and the database

### The sample through the study waves

The ECPBHS originates from the European Youth Heart Study (EYHS; e.g., Andersen *et al*., 2006); in Estonia it was conducted in 1998 and 1999. Each country participating in the EYHS aimed to include 1000 children, 500 aged 9 and 500 aged 15, but to account for incomplete data, the Estonian team decided to sample 600 + 600 children. The selected age groups reflected key stages of sexual maturation: 9-year-olds were typically prepubertal, while 15-year-olds were in the final stages of puberty (Harro M *et al*., 2001). The Estonian sample involved a homogeneous sample of Caucasian participants representing both sexes and the main ethnic groups in Estonia (Estonian and Russian), as well as urban and rural populations. The number of children in each category was determined based on the distribution of 9- and 15-year-olds in Tartu County, as per local statistics.

The school was used as the primary sampling unit. Headmasters from 54 of the 56 schools in Tartu County, which included 9- and 15-year-old students, agreed to participate. The minimum expected number of children per age group per school was set at 25. A random sample of 25 schools was drawn using cluster sampling, stratified by location (urban or rural), age group, and language of instruction (Estonian or Russian), with probability proportional to the number of students of the respective age groups in the school. All students in grades 3 and 9 at the selected schools were invited to participate. Of the 1486 invited participants, 79.1% (*n* = 1176) consented and were enrolled in the study. In 2001, the decision was made to conduct a follow-up of the older cohort, until they still attend their schools. The short preparation period together with the reluctance of schools to grant free time in spring just before exams reduced the number of participants, therefore an additional 62 individuals from other schools were included in the study.

**Figure 1.**
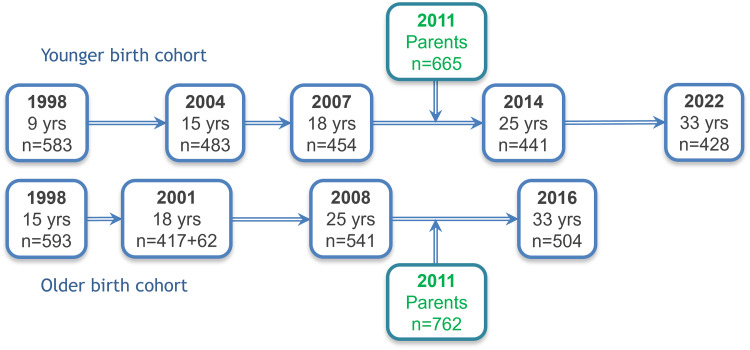
Timelines of the Estonian Children Personality Behaviour and Health Study portrayed schematically. For more detail, see Table 1.

This original sample, consisting of two birth cohorts, was followed longitudinally; all participants have been assessed at ages 15, 18, 25, and 33 (Figure 1).

At ages 15 and 18, participants were mostly still found at the schools they had attended during the first study wave. Those no longer there, an invitation letter was sent to their home address, which was obtained from other participants in the study or the population register, or they were contacted by phone. At ages 25 and 33 (and in the parents’ study wave), home addresses were requested from the Estonian National Registry. Invitation letters were sent to the participants’ electronic and home addresses, collected in former study waves, or from their parents or from other ECPBHS participants. If the letter garnered no response, the participants received a call on their personal mobile phones. Non-responders were subsequently contacted via social media (Orkut or Facebook) or work phone and, before year 2016, home visits were also conducted. These procedures helped to achieve low attrition (Table 1).

The parents of the birth cohort participants were engaged from the outset by completing various questionnaires about their child and family. However, between 2011 and 2013, mothers and fathers were invited to join the study and data were collected about the parents themselves. In total, 1416 parents (581 fathers and 835 mothers, with mean age at the laboratory visit 54.1 ± 6.5 and 52.0 ± 5.8, respectively) were included.

**Table 1.** Participation in the data collection waves of the Estonian Children Personality Behaviour and Health Study by birth cohort and gender

		9 years old	15 years old	18 years old	25 years old	33 years old	Parents
Birth cohort		1998–1999	2004–2005	2007–2008	2014–2015	2022–2023	2011–2013
Younger cohort	*n* (%) gender mean age (± S.D.)	583 M 278; F 305 9.5 ± 0.5	483 (82.8%) M 222; F 261 15.3 ± 0.7	454 (77.9%) M 202; F 252 18.3 ± 0.5	441 (75.6%) M 195; F 246 25.3 ± 0.5	428 (73.4%) M 195; F 233 33.6 ± 0.6	665 M 279; 51.6 ± 6.4 F 386; 49.0 ± 5.1

			1998–1999	2001–2002	2008–2009	2016–2017	2011–2013
Older cohort	*n* (%) gender mean age (± S.D.)		593 M 260; F 333 15.4 ± 0.6	417 (70.3%) + 62 additional participants M 208; F 271 18.4 ± 0.9	541 (82.6%) M 230; F 311 24.7 ± 0.7	504 (76.9%) M 211; F 293 33.5 ± 0.7	762 M 308; 56.5 ± 5.8 F 454; 54.6 ± 5.1

*n*, number of participants; M, male; F, female; % - proportion of the total sample at follow-ups; S.D., standard deviation.

The total number of the ECPBHS parents is 1416: six fathers and five mothers have a child in both birth cohort samples. The numbers in the table represent the child-associated information on parents themselves.

### Data collection procedure

Children were picked up at their school in the morning and taken to the laboratory. Adults came on their own. Typically, fasting participants arrived between 8:00 and 9:00 a.m. They were instructed not to eat or drink that morning. First, blood samples were collected. After a light breakfast blood pressure was measured (before drinking coffee), anthropometric measurements were taken and an aerobic fitness test was conducted. Participants completed questionnaires, attended the diet interview and performed computer tasks in a variable order.

When the participants were 9, 15 or 18 years old, a parental questionnaire consisting of three sections (A, B, and C) was administered. Section A was completed by a parent with information on the child’s lifestyle, health and risk behaviours. Section B was completed by the mother, and Section C by the father. These sections included questions concerning the parents’ health, education, occupation, and living conditions, as well as the child’s health and personality. A few psychometric instruments were also administered to class teachers. In 2011–2013, the parents themselves completed the core set of the questionnaires administered to their children as adults. They also took part in the dietary assessment, measurement of anthropometric and physiological parameters and physical activity, and biosamples were collected.

At all study waves, participants completed a self-administered questionnaire concerning their living conditions, mental and physical health, socioeconomic background, and trusted persons. The questionnaire also included sections on risk behaviours and physical activity. Several additional instruments were used to assess personality and behavioural characteristics.

The set of instruments used for data collection expanded over the years and, although data on key indicators were gathered in every wave, some variation of content exists between the waves, owing to involvement in European research consortia with their specific objectives, or the conceptual evolution among the EstChild team. The volume of questionnaires thus became burdensome to be completed during the laboratory session. Consequently, the material was divided into three packages A, B, and C: Package A was mailed to the participants and completed prior to the laboratory visit, B during the visit, and C was taken home and returned subsequently together with the accelerometer, that had been distributed during the laboratory visit.

Measures and biosamples collected from the ECPBHS target subjects are listed in Table 2, and detailed information on instruments is provided in Supplementary Tables 1 and 2. Parents of the ECPBHS children provided material on all the main constructs, with the exception that aerobic fitness was not assessed (Supplementary Table 3).

**Table 2.** Data collected in the Estonian Children Personality Behaviour and Health Study since 1998. More detail on the measures, instruments and equipment used in each study wave can be found in Supplementary Tables 1 and 2. Most of these measures and constructs were also addressed in the parents’ data collection wave (Supplementary Table 3)

Measure or construct	Method of measurement	Age or study wave, and other pertinent comments
Anthropometric and physiological parameters	Objective measurement of height, body mass, waist and hip circumference, five skinfolds, blood pressure	In all study waves
Pubertal status	Observation	At age 9 and 15
Biosamples for cardiometabolic and psychiatric biomarkers (blood components; saliva; DNA; RNA)	Clinical biochemistry Radioenzymatic assay Single SNP genotyping Genome-wide assays Epigenetic, including epigenome-wide assays	Biosamples collected in all study waves; sample processing and measurements continue
Health and well-being	Questionnaire	In all study waves At age 9 to 18, additional information from parents
Incidence of diseases	Questionnaire	In all study waves At age 9 to 18, additional information from parents
Physical activity	Questionnaire Accelerometry-based measurement	Age-adjusted In all study waves At age 9 to 18, additional information from parents
Aerobic fitness	Maximal power output test on a veloergometer	In all study waves
Diet	Questionnaire Diet diary, followed by a diet interview Nutrient and food group intake quantification based on the national food composition database	In all study waves The diet record period in the diary was increased from the initial 24 h to 72 h
Socio-economic situation and lifestyle	Questionnaire	In all study waves, age-adjusted; At age 9 to 18, additional information from parents
Personality: Big Five (BF); Affective Neuroscience (ANPS)	Questionnaire	BF in all study waves, measures varied and were adjusted for age; Self-reports from age 15, parental report at age 9, 15, and 18, teacher report at age 9 and 15 ANPS in adulthood
Psychological traits: impulsivity, aggressiveness, inattention and hyperactivity, reward sensitivity	Questionnaire	In all study waves, instruments varied by age For ADHD traits, information also from parents and teachers (age 9, 15, and 18)
Life history of aggression	Interview	Younger cohort at age 25, older cohort at age 33
Cognitive abilities	Raven Progressive Matrices (RPM) Computer-based tests of processing speed, memory updating, cognitive flexibility and impulsive tendencies	Different sets of RPM at different ages Tests varied by study wave
Traffic behaviour	Questionnaire	Measures were age-adjusted
Alcohol, tobacco, and illicit drug use	Questionnaire	In all study waves, measures varied and were adjusted for age Additional information from parents at age 9 to 18
Mood measures: anxiety, depressiveness, affect intensity, fears, self-esteem	Questionnaire	Depressiveness and anxiety in all study waves, other measures from age 18 or 25
Life satisfaction	Questionnaire	Measured at ages 25 and 33
Psychosocial environment and life events	Questionnaire	In all study waves, age-adjusted; At age 9 to 18, additional information from parents
Work-related psychosocial factors	Questionnaire	At age 25 and 33 in both study cohorts
Eating disorder symptomatology	Questionnaire	From age 15 to female participants
Psychiatric disorders	Interview (MINI)	At age 25; current and lifetime incidence

Participation in the study was voluntary in each wave, and participants were allowed to refuse any procedure they found uncomfortable. Written informed consent was obtained from all participants prior to enrolment; in the case of minors, consent was also provided by their parents. The study was conducted in accordance with the Declaration of Helsinki. Ethical approval for each study wave was granted by the Ethics Review Committee on Human Research of the University of Tartu.

### Anthropometry, and physical health objectively and subjectively

Height was measured with a stadiometer (Tanita HR 001; Amsterdam, TANITA Europe B.V.) to the nearest 0.5 cm, body mass was initially measured using a calibrated beam balance, later with an electronic scale with the subject in light clothing to the nearest 0.1 kg, and body composition assessed (Omron BF300, Kyoto, OMRON Matsusaka Co. Ltd., or BC-420MA; Amsterdam, TANITA Europe B.V.). Waist and hip circumferences were measured with a metal anthropometric tape. Waist circumference was measured midway between the lower rib margin and the iliac crest at the end of gentle expiration. Hip circumference was measured as the largest circumference of the buttocks. Skinfolds were measured on biceps brachii, triceps brachii, subscapular, suprailiac, and medial calf with a Harpenden calliper (Baty International, Burguess Hill, U.K.) (Lohman *et al*., 1991). All measurements were taken twice, and the mean value was calculated (Ortega *et al*., 2007). Based on anthropometric indicators, body mass index (BMI = body weight/height^2^ [kg/m^2^], waist-to-hip ratio (WHR) and the sum of five skinfold thicknesses were calculated.

Resting blood pressure was measured on the upper arm with an automatic blood pressure monitor (Dinamap Compact; Critikon L.L.C. Tampa, FL, U.S.A.) five times at 2-min intervals, from which the average systolic and diastolic blood pressure values were calculated. In the original data collection wave, lung function was assessed with the Vitalograph 2170 (Vitalograph Ltd. Maides Moreton, Buckingham, England) spirometer.

The pubertal stage was assessed in a private room by an experienced medical researcher of respective gender, following a brief observation, in 9- and 15-year-old subjects (Tanner & Whitehouse, 1976).

As an indicator of subjective physical and psychological health, and general well-being, a measure of self-rated health was taken on a 5-point scale (Pärna & Ringmets, 2011) health questionnaire also recorded the incidence of acute diseases, accidents, injuries, and health complaints, as well as lifetime chronic conditions.

### Physical activity and aerobic fitness

Physical activity was assessed both by structured questionnaires and objectively by using accelerometry (Kiive *et al*., 2025). Participants wore the accelerometer (Caltrac, Hemokinetics, Madison, WI, U.S.A. or ActiGraph GT1M or GT3X, Monrovia, CA, U.S.A.) on their right hip. Reliable accelerometer data required at least eight hours of daily recording for a minimum of four days, including two weekend days. The average data from the recorded days was calculated for total physical activity (counts per minute), sedentary time, and light, moderate, and vigorous activity. Additional information concerning the training status, participating in training groups and attitude towards training, was also recorded. In addition to self-assessment, parents also evaluated their child´s physical activity via questionnaire.

Aerobic fitness was assessed using a cycle ergometer with progressively increasing load until exhaustion (Muntaner-Mas *et al*., 2021). In the earlier study waves, the Monark 839E (Monark Exercise, Vansbro, Sweden) was used, while later waves utilised the Tunturi T8 (Tunturi OY, Turku, Finland) or Ergoselect 100 (Ergoline Gmbh, Bitz, Germany). Each test began with a 3-min warm-up. In the initial study wave, the starting work rate was set at either 20 or 25 W, depending on whether the subjects weighed less than or more than 30 kg, respectively. In subsequent waves, the starting work rate for females was set at 40 W with subsequent increases of 40 W, while for males, the initial workload was 50 W, increasing by 50 W. Throughout all study waves, each workload was maintained for three minutes. The bike ergometer allowed participants to choose a comfortable pedalling cadence within a range of 60 to 80 revolutions per minute (rpm). Heart rate (HR) was continuously monitored during the test using a Polar Vantage HR monitor (Polar Electro, Kempele, Finland). Criteria for exhaustion were defined as either a heart rate exceeding 185 beats per minute, the ability to maintain a pedalling rate of at least 30 rpm, or the subjective judgement of the observer. Aerobic fitness was calculated as maximal work output (Wmax) and further adjusted for body weight (Wmax/kg). Maximal power output was determined for each individual using the formula: W1 + (W2 × t/180), where W1 is the work rate at the fully completed stage, W2 is the work rate increased at the final incomplete stage, and t is the time in seconds at the final incomplete stage. After exercise, participants rated their perceived exertion (Borg, 1998).

### Diet diary and interview

Dietary assessment included questionnaires, diary and an interview (Matrov *et al*., 2022). Participants were instructed to complete a food record at home prior to the study day (covering a period from 24 to 72 h, as the study progressed). For participants in the younger cohort, who were initially 9 years old, parental assistance was provided when necessary to ensure accurate reporting.

On the study day, a face-to-face interactive interview was conducted. The food record data were validated against the interview responses, and any discrepancies were discussed with the participants. Portion sizes not specified in the food records were estimated using pictures of portion sizes (Haapa *et al*., 1985). Dietary intake over several days was averaged to calculate the mean food consumption.

Nutrient and food group intake was analysed using the Finnish Micro-Nutrica Nutritional Analysis platform, adapted to include Estonian foods, and the evidence-based food composition Estonian database NutriData, maintained by the National Institute for Health Development (Tallinn, Estonia). A food frequency questionnaire was completed by the participants at home beforehand and brought with them on the study day. This questionnaire addressed the frequency of consumption of certain food groups and food items, including e.g., caffeine.

### Questionnaires on socio-economic situation and lifestyle

Questions on socioeconomic background, living conditions and lifestyle varied across study waves; they were age-appropriate and became more specific as participants grew older. For participants aged 9–18, parents helped to provide information. For example, the questionnaire for participants aged 9–18 included sections on school-related topics, such as the relationships with teachers and classmates, academic progress, and the overall school climate (Vaht *et al*., 2016). It included questions about eating habits and attitudes, physical activity, sports participation, and commuting (e.g., to school). Several items addressed hobbies, friends and pets, television viewing and video gaming habits, and, in later waves, internet use. Parental education level, household income, living conditions, and perceived socioeconomic status (SES) compared to peers (ranging from ‘poor’ to ‘among the wealthiest in the country’) were queried (excluded at age 9).

At age 25 and 33 the cohorts faced questions about the level of education, total household income, and self-reported SES assessment compared to peers, living conditions, parenthood, and occupational classification.

### Personality assessment: comprehensive models

Personality data based on the Five-Factor Model (Neuroticism, Extraversion, Openness, Agreeableness, and Conscientiousness) were reported at ages 9, 15, and 18 by the mother and the father, and from age 15 also self-reported, using a variety of instruments: the Estonian Brief Big Five Inventory (Laidra *et al*., 2006), the Estonian version of the NEO-PI-R (Kallasmaa *et al*., 2000), the Short Five (Konstabel *et al*., 2012) or, most commonly and in the most recent waves, the semantically simplified EE.PIP-NEO (Mõttus *et al*., 2006).

The more recent data collection waves used an adaptation (Harro *et al*., 2019) of the short version of the Affective Neuroscience Personality Scale ANPS (Davis *et al*., 2003), which is a self-report instrument constructed bottom up to correspond to the activity in neural circuits underlying basic emotive systems as defined in animal research (Panksepp, 1998). It comprises scales termed ANGER, FEAR, SADNESS, SEEKING, CARE, and PLAY.

### Psychological traits: impulsivity, aggressiveness, attention deficit, hyperactivity, reward sensitivity

Hyperactive behaviour and concentration difficulties were assessed from age 9 by class teachers and parents (Kiive *et al*., 2010) using the 7-point Hyperactivity Scale (af Klinteberg & Oreland, 1995). In addition, data on ADHD symptoms in adolescents were collected using teacher- and parent-rated Swanson, Nolan and Pelham Questionnaire IV (SNAP-IV; Swanson, 1992). As young adults, participants filled in the World Health Organisation Adult ADHD Self-Report Scale symptom checklist (ASRS; Kessler *et al*., 2005). Because the wording of the ASRS items is linguistically complex, the ADHD symptomatology of both cohorts at age 33 was also assessed using a measure specifically constructed for this purpose, featuring simple and easily understandable statements (Simple ADHD). Furthermore, at age 33, inattention and concentration difficulties were assessed using the Attention Control Scale (ATTC; Derryberry & Reed, 2002), a self-report measure evaluating two key components of attention: focusing and shifting.

Facets of impulsivity (Fast Decision Making, Excitement Seeking, Disinhibition, and Thoughtlessness) were assessed by self-report from ages 15 to 33 using the Adaptive and Maladaptive Impulsivity Scale (AMIS). The AMIS is an original composite instrument based on the Dickman Impulsivity Inventory (Dickman, 1990) and impulsivity-related subscales of the NEO Personality Inventory. Fast Decision Making and Excitement Seeking can be combined to form the Adaptive Impulsivity score, whereas Disinhibition and Thoughtlessness comprise Maladaptive Impulsivity (Paaver *et al*., 2006). In addition, the Barratt Impulsiveness Scale, 11th version (BIS-11; Patton *et al*., 1995), was employed.

Aggressive behaviour was assessed across the study. At ages 9, 15, and 18, class teachers who had known the child for at least three years rated aggression using the Aggressiveness subscale of the 7-point Hyperactivity Scale (af Klinteberg & Oreland, 1995). In adulthood, aggression was self-reported using the Buss-Perry Aggression Questionnaire (BPAQ; Buss & Perry, 1992). BPAQ specifies Physical Aggression, Verbal Aggression, Anger, and Hostility. Additionally, the Aggressive Provocation Questionnaire (APQ; O’Connor *et al*., 2001), based on Frijda’s modular theory of emotions (1988; 2017), was used to assess aggressive tendencies in provided real-life situations.

The Illinois Bully Scale (IBS; Espelage & Holt, 2001) comprises three dimensions (Bully, Fight, and Victim) and, although originally designed for 8–18-year-olds, was used here as a retrospective measure. Participants also completed peer ratings of classmates they remembered well; scores were averaged when multiple ratings were available. One original instrument (KVLS-KAKK) was designed for the purpose of research on types of aggressiveness less studied in the general population, but it has not yet been properly described.

Reward sensitivity was assessed using the original Reward Openness and Insatiability Scale (ROIS; Pulver *et al*., 2020a) that will be discussed below.

### Cognitive abilities and computer-based tasks

As a paper and pencil test, Raven’s Standard Progressive Matrices (RPM) (Raven, 1998), standardised on the Estonian population (Lynn *et al*., 2002), was used to measure the nonverbal intellectual abilities. No time limit was applied.

In specific study waves, a variety of computer-based experiments were conducted to measure processing speed, memory updating, cognitive flexibility and impulsive tendencies in the participants. These included the Visual Comparison Test (VCT; based on Dickman & Meyer, 1988; Paaver *et al*., 2007), the Wisconsin Card Sorting Test (WCST; Grant & Berg, 1948), the Stop Signal Task (SST; based on Logan *et al*., 1997; Reif *et al*., 2011), a 2-back working-memory updating task using simple schematic facial expressions (Tamm *et al*., 2021), the CogShift task, originally based on the attention-switching paradigm described by Ravizza and Ciranni (2002), and a visual discrimination task modelled on Forster and Lavie’s low-load paradigm (Forster & Lavie, 2015; Tuvi *et al*., 2020).

### Behaviour in traffic

The traffic safety questionnaire (Eensoo *et al*., 2007; Luht *et al*., 2018) was introduced in year 2004 and was age adjusted (e.g., schoolchildren were questioned about ‘frequency of using pedestrian crossings on the way to school’, and young adults about ‘using a mobile phone while driving’).

Participants who had driven a car in the year prior to the study have completed the Driver Behaviour Questionnaire (DBQ, Reason *et al*., 1990; Tokko *et al*., 2022), Driver Anger Scale (DAS, Deffenbacher *et al*., 1994; Tokko *et al*., 2023) and Driver Skill Inventory (DSI, Lajunen & Summala, 1995; Eensoo *et al*., 2010). The majority of participants have consented to anonymized accession to their objective traffic records.

### Substance use

Alcohol and tobacco use were self-reported in all study waves. The questionnaires addressed, for example, the age of initiation, quantity, and frequency of alcohol and tobacco use, as well as the preferred type of alcohol or tobacco product, and were adapted to be age-appropriate. At age 33, the Alcohol Use Disorders Identification Test (AUDIT, Saunders *et al*., 1993) was applied, and the Fagerström test (Heatherton, 1991) to detect possible nicotine dependence.

### Mood measures

The State–Trait Anxiety Inventory subscales (STAI-S and STAI-T; Spielberger *et al*., 1983) has been administered since age 15. Depressive symptoms were assessed using the Montgomery-Åsberg Depression Rating Scale (Montgomery & Åsberg, 1979) and the Beck Depression Inventory (Beck *et al*., 1961).

We compiled a list of fears that included items known to be common sources of phobias and items that had been described as the most common fears in adolescents and youth from previous literature. The respondents were asked to assess how much they fear 18 items on a 5-point scale ranging from 0 = *not scared* to 4 = *very scared* (Tulviste *et al*., 2015).

The Rosenberg Self-Esteem Scale (Rosenberg, 1965; Pullmann & Allik, 2000) assessed an overall positive or negative attitude toward oneself. Global cognitive evaluation of life satisfaction was made with the Satisfaction with Life Scale (Diener *et al*., 1985).

### Psychosocial environment and life events

At its beginnings, ECPBHS applied a questionnaire used by sociologists of childhood. This served as the source of the development of the Tartu Family Relationships Scale (TFRS; Paaver *et al*., 2008). The TFRS measures four dimensions of family functioning: closeness, support, misprise, and abuse, which can be combined into two higher-order scales: warmth (Closeness and Support) and maltreatment (Misprise and Abuse). In two study waves, the Parental Acceptance–Rejection/Control Questionnaire (PARQ/Control; Tulviste & Rohner, 2010) was used for mother–child relationship, and when the adolescents reached adulthood, at ages 25 and 33, they were asked about their current relationships with their mothers and fathers. In addition, 25- and 33-year-olds who reported being in a stable cohabiting romantic relationship, either currently or in the past, described their relationship with their partner. For this purpose, an instrument based on the TFRS was used, with the questions adapted to assess a partner relationship.

History of stressful life events (SLE) was self-reported by participants (Laas *et al*., 2014b). The list of adverse life events varied across study waves and consisted of 10–17 (dependent on the study wave) stressful events including parental death, parental divorce or separation, absence of both parents, familial unemployment, financial difficulties and poverty, poor living conditions, poor health and chronic diseases, serious illness of a family member, death of a close relative, trauma, fear of school, bullying at school and humiliation at home. At ages 25 and 33, recent stressful life events encountered in the five preceding years were self-assessed. The checklist consisted of 14 personal SLE-s involving divorce/termination of any romantic relationship, separation from a significant other, serious conflicts with husband/wife/partner, having been assaulted, major financial trouble, difficulties with residence, serious illness or injury, being laid off or sacked from work, trouble with the law, having been robbed, serious problems at work, birth of a child, traffic accident, or other accident considered serious. Data were also collected on SLE-s related to significant other/family members, such as conflicts with significant other/family member and personal crisis, death or serious illness of significant other/family member. The events were recorded as dichotomous variables (present or absent) and then counted to determine the number of experienced adverse life events.

In adulthood, the Copenhagen Psychosocial Questionnaire (COPSOQ; Kristensen *et al*., 2005) medium-length version was used to assess psychosocial factors at work, stress, and well-being.

### Disordered eating

Eating behaviour and attitudes were assessed in female participants from age 15 onward by the Eating Disorders Inventory-2 (EDI-2; Garner, 1991; Podar *et al*., 1999), a self-report questionnaire designed to assess eating disorder psychopathology.

### Clinical interview for lifetime incidence of psychiatric disorders and aggressiveness

The Mini-International Neuropsychiatric Interview (M.I.N.I.5.0.0; Sheehan *et al*., 1998; Estonian version Shlik *et al*., 1999) was used to screen for current and lifetime psychiatric disorders. M.I.N.I. 5.0.0 is a structured psychiatric interview developed to diagnose *DSM-IV* and *ICD-10* mental disorders. A diagnostic assessment was conducted by experienced clinical psychologists in both study cohorts at the age of 25.

An interview of Life History of Aggression (LHA) was conducted by an experienced clinical psychologist. The aggression subscale of this measurement tool for lifetime aggressive behaviour (Coccaro *et al*., 1997) was used to assess dimensions of aggression, including adult frequency of temper tantrums, general fighting, specific physical assault, specific property assault, and verbal assault.

### Biosamples, biochemical measures and genotyping

Biosamples were collected at each study wave, prepared for a variety of assays, and have been stored in a biobank. Cardiovascular risk indicators, including triglycerides, cholesterol, HDL-cholesterol, and LDL-cholesterol, have been measured in an accredited laboratory. Platelet monoamine oxidase has been measured radioenzymatically (Harro M *et al*., 2001). Several hypothesis-based and hypothesis-free genetic and DNA methylation analyses have been conducted (see the following sections). As expected from such a sample, each genotype has been in Hardy–Weinberg equilibrium.

The perspective-opening findings that have energed from the ECPBHS are summarized in Table 3. Below these results have been put into context.

**Table 3.** A selection of perspective-opening findings that have emerged in the ECPBHS/EstChild and are briefly discussed in the following sections

Fitness and body fat already at age 18 related to lipids, glucose, insulin, and blood pressure
Fitness independently contributes to the metabolic risk score
Decline in early fitness negates any later reduction in adult metabolic risk
Low childhood cardiorespiratory fitness associated with later ADHD symptoms
NPY gene associations with health and behaviour age- and period-dependent
Platelet MAO activity associated with risk-taking choices, also in females
Association of cholesterol measured in childhood with adult impulsivity
5-HTTLPR genotype associated with personality development
5-HTTLPR genotype and life events interaction in eating disorder symptoms
Novel inner structure for reward sensitivity with distinct associations with personality and metabolism
Gene × environment interactions shaping adaptive vs. maladaptive impulsivity
Orexin receptor genotype associated with aggressiveness
Hypothesis-free epigenome search reveals a novel gene related to aggressiveness
Distinct gene × environment interaction dependent paths to alcohol use disorder
Gene × environment interactions largely sex/gender dependent
Functional gene variants associated with educational attainment
Birth cohort effects appear in gene × environment interactions

## Cardiovascular risk factors in ECPBHS and their relevance to neuroscience

Physical fitness, physical activity, and metabolic health are closely related. Low cardiorespiratory fitness is associated with insulin resistance, poor lipid metabolism, and excess body fat (Ortega *et al*., 2018). Conversely, metabolic dysfunction can impair exercise capacity and diminish the body’s ability to sustain cardiovascular fitness. Cardiorespiratory fitness refers to the capability of the circulatory and respiratory systems to supply oxygen to skeletal muscles for energy production during physical activity (Raghuveer *et al*., 2020). Research indicates that metabolic risk factors can track from childhood and adolescence into adulthood (Juhola *et al*., 2011). Furthermore, evidence suggests that being physically fit, regardless of body fat levels, can positively influence metabolic markers in young adults (Sacheck *et al*., 2010; Tur-Boned *et al*., 2025).

In the ECPBHS birth cohorts, both fitness and body fat were related already at age 18 to blood lipids, glucose levels, insulin, and blood pressure, and fitness independently contributed to the metabolic risk score (Lätt *et al*., 2018). However, high cardiorespiratory fitness in adolescence, if followed by a decline in fitness during adulthood, does not yield benefits compared to consistently low cardiovascular fitness from age 15 to 33, while improving cardiorespiratory fitness from adolescence to adulthood can lower metabolic risk (Mäestu *et al*., 2020).

Metabolic disturbances are characteristic of a variety of neuropsychiatric disorders, and physical activity has successfully served as an adjunctive treatment (Vancampfort *et al*., 2025). Attention-deficit/hyperactivity disorder (ADHD) is a neurodevelopmental disorder that begins in childhood and often persists into adulthood. It has been associated with obesity (Cortese & Tessari, 2017), diabetes and hypertension (Chen *et al*., 2018). Different aspects of physical fitness are associated with ADHD in young adults (Jeoung, 2014) and interventions aimed at increasing physical activity have been shown to improve attention and reduce impulsivity in individuals with ADHD (Vancampfort *et al*., 2025). The ECPBHS was the first study showing with longitudinal design that lower cardiorespiratory fitness in childhood is associated with increased, indeed doubling, ADHD symptoms during adolescence, even after adjusting for baseline ADHD symptoms (Muntaner-Mas *et al*., 2021). This association has recently been confirmed at the diagnostic level in a large national sample of 1.9 million participants in Taiwan (Chiang *et al*., 2024).

An intriguing case of apparent non-association with body composition and metabolic function is presented by the neuropeptide Y (NPY) gene. NPY, a 36-amino acid neuropeptide (Tatemoto *et al*., 1982), much studied in the context of anxiety, addiction and depression (Heilig & Widerlöw, 1995; Wu *et al*., 2011; Thorsell & Mathé, 2017), is a most potent orexigenic stimulus (Kokot & Ficek, 1999) that leads to an increase in fat mass and body weight (Stanley *et al*., 1986). Also, *NPY* expression is increased in adipose subjects (Sitticharoon *et al*., 2013), and physical activity has an impact on NPY gene expression that may relate to its antidepressant properties (Melas *et al*., 2013). Nevertheless, hypothesis-free searches in the genome have not identified *NPY* as a risk gene for any of the plausible candidate conditions, and the evidence for association of in vitro functional variations (Kallio *et al*., 2001; Zhou *et al*., 2008) in body composition is controversial. When the ECPBHS cohorts were analysed longitudinally for *NPY* variants, all examined genotypes were found as associated with changes in metabolic markers and with blood pressure (Katus *et al*., 2021). Age appeared as a modifying factor, possibly owing to lifestyle changes in transition from childhood to adulthood. Indeed, animal experiments have suggested that the effect of the orexigenic stimulus by NPY on feeding behaviour can be strongly dependent on a complex sequence of preliminary behaviours (Woods *et al*., 1998), and thus conceivably contingent on environmental factors.

## Classic psychiatric risk markers: resurrection of platelet MAO and new thoughts about low cholesterol

Human platelets express the monoamine oxidase B isoenzyme (MAO-B, platelet MAO), that was long ago proposed as a marker of general psychiatric vulnerability (Buchsbaum *et al*., 1976). The finding that tobacco smoking has a limited but dose-dependent inhibitory effect on MAO activity (Fowler *et al*., 1996) depressed interest in this measure. (The measurement is also technically demanding.) Nevertheless, the body of evidence has indirectly suggested that, despite smoking being prevalent in patients and in socially deviant groups, platelet MAO activity is the most reliable biological correlate of impulsivity-related behaviours, especially if coexistent with other, independent risk factors (Oreland, 2004). For example, platelet MAO activity was lower in passenger car drivers who had been caught driving drunk (Eensoo *et al*., 2004) and in patients with traumatic spinal cord injury (Sabre *et al*., 2016); low platelet MAO was particularly prevalent in youngsters who had repeatedly committed criminal acts if this co-occurred with high psychopathy scores (Alm *et al*., 1996). Incidentally, the initial ECPBHS paper reported on platelet MAO activity, confirming for the first time in a birth cohort representative sample that males have, on average, lower enzyme activity than females (Harro *et al*., 2001). An important finding made in longitudinal observation was that platelet MAO activity is predictive of initiation of smoking, whereas both lower and higher than average enzyme activity related to smoking (Harro *et al*., 2004). Indeed, the work of Alm et al. (1996) had shown the overrepresentation of subjects with both low and high platelet MAO activity among juvenile delinquents, highlighting the point that both low and high platelet MAO levels, reflective of lower and higher central serotonergic capacity (Fahlke *et al*., 2002; Eriksson *et al*., 2006; Harro *et al*., 2025), are risk factors for suboptimal behavioural choices, possibly owing to early developmental impact of serotonin (Harro & Oreland, 2016; Shah *et al*., 2018). A twin study has revealed that when the analysis is corrected for smoking, platelet MAO activity can be positively associated with neuroticism (Kirk *et al*., 2001), which is also a risk factor not only for commencing smoking (Byrne *et al*., 1995) but more generally of deviant brain responses and behaviour under pressure (Zhu *et al*., 2023; Ziegler *et al*., 2024; Fioravanti *et al*., 2025).

One controversial aspect in this regard has always been the very unreliable association of low platelet MAO activity with substance use disorders (apart from Type 2 alcoholism), given their link to risk-taking and antisocial conduct. In the ECPBHS males, low platelet MAO activity was indeed associated with higher prevalence of illicit drug experience, and with earlier debut, but this association was even stronger in subjects who had tried the substance only once (Sakala *et al*., 2022). This observation explains the missing link between illicit drug use disorders and low platelet MAO activity, but the underlying mechanism of why the low MAO subjects less often progress from initiation to regular use remains to be clarified. This could relate, e.g., to higher novelty-driven behavioural strategies, less pleasurable (early) drug experience or lower sensitisation of the brain salience-attributing system by the drugs.

Low serum levels of cholesterol is another much-studied biomarker in psychiatry, often associated with impulsive action and suicide (e.g., Engelberg, 1992; Wu *et al*., 2015; Khosravani *et al*., 2025), but not previously investigated with longitudinal design. Taking advantage of the ECPBHS database, we have assessed the link between impulsivity measured in young adulthood (age 25) and cholesterol levels measured at four time points, starting from age 9. In this sample, the correlation between impulsivity self-reports and cholesterol levels was stronger, the earlier the serum sample was collected (Tomson-Johanson *et al*., 2020). This unexpected finding suggests that the association between cholesterol levels and impulsivity is the reflection of developmental processes at early age.

## The 5-HTTLPR in the ECPBHS

5-HTTLPR, denoting the serotonin transporter gene linked polymorphic region (Heils *et al*., 1996), is almost a notorious gene variation, as it has become the most studied target in the ‘candidate gene’ studies of psychiatric disorders and a variety of behaviours, and brain structure and function. The very strong association of the short (s) versus long (l) allele (meaning the extent of tandem repeats) in the putative promoter region with neuroticism, harm avoidance and anxiety disorders in the original study (Lesch *et al*., 1996), however, immediately raised the question of what is the evolutionary meaning of such a high frequency of the allegedly neuroticism-promoting s-allele, present in the majority of Europeans and in the vast majority of Japanese. Several subsequent studies have not replicated the original association. While the findings on the Dunedin Birth Cohort demonstrated that the s-allele, and especially the s/s-homozygocity, contributed strongly to depression and suicidality only in the context of stressful life events (Caspi *et al*., 2003), even these results were not easily replicable. The sheer number of research papers reporting statistically significant associations of 5-HTTLPR with a wide variety of behaviours and measures suggests that this genetic variation may bear important functional consequences at a very deep layer of neural processes, with huge dependency of the observable phenomena on the multiple developmental steps. The ECPBHS sample was explored for 5-HTTLPR after successful replications of the original finding (Hariri *et al*., 2002) that the amygdala activation is higher in carriers of the 5-HTTLPR s-allele. Importantly, an analogous polymorphism, present in rhesus macaques, was found associated with higher frequency or longer duration of exhibiting fear and anxiety related behaviours in several non-human primate populations (Bennett *et al*., 2002; Howarth *et al*., 2023). We observed higher Big Five neuroticism in the 5-HTTLPR s/s-homozygous participants, but also lower openness to experience, agreeableness and conscientiousness at age 9 as reported by parents; extraversion was not associated with the 5-HTTLPR (Harro *et al*., 2009). At age 15, parental reports no longer differed by genotype, but the s/s-homozygous subjects self-reported higher neuroticism. By age 18, this difference had also disappeared. In contrast, as the children aged, the 5-HTTLPR genotype effect emerged in terms of experimenting with intoxicants: this measure increasing with age, the 5-HTTLPR s/s-homozygocity more frequently associated with smoking, alcohol intake and use of illicit drugs (Merenäkk *et al*., 2011; Vaht *et al*., 2014). Hence, in a representative sample, the 5-HTTLPR genotype is associated with personality and behaviour, while the associations follow the developmental curve. More recently, ongoing brain imaging studies have shed further light on the impact of the 5-HTTLPR to structure and function of limbic networks, and shown it to be complex (e.g., Hariri *et al*., 2006; Kirchner *et al*., 2023: Klöbl *et al*., 2025) and experience-dependent (Ancelin *et al*., 2021), which may lead to variable results between studies on highly divergent samples. Nevertheless, response of the neurohormonal stress axis diverges by genotype (Sun *et al*., 2020; Kuhn *et al*., 2021). The 5-HTTLPR s-allele carriers appear as having a higher sensitivity to stress, but also higher capability to recover, adding a time dimension to the gene × environment (G×E) interaction studies (Delli Colli *et al*., 2022). Thus, with regard to compromising mental health, the 5-HTTLPR s-allele may serve as an amplifier of more causal factors, such as, in the ECPBHS sample, enhancing anxiety and bulimia in subjects with binge eating (Akkermann *et al*., 2010). Fittingly with this example, the l/l-homozygocity of the 5-HTTLPR is associated with stronger responses to high-calorie food in cognitive control brain areas, indicating better capacity to resist the food reward (Markus and Keulers, 2025).

## Gene × environment interactions: from family relationships to birth cohort effects

Gene variants interact with environmental factors in the development of the nervous system, in the formation of behavioural traits, and in the pathogenesis of psychiatric disorders – but how? We know something about the structure of heredity, but not much about the interrelationship of all things that can be interpreted as environmental impacts. What is defined as adverse life events may differ in important ways with regard to their power, duration, and timing. ECPBHS has a rich collection of environmental data, which indicate that common gene variants often interact with the sum of adverse life events quite differently in comparison with the perceived family environment.

While not the first functional polymorphism shown to interact with environmental measures, 5-HTTLPR was made the spearhead case by the report of Caspi *et al.* (2003). Also the monkey variant of 5-HTTLPR is the subject of G×E interactions, the s-allele carriers being more sensitive to adverse rearing conditions in terms of brain development (Schoenfeld *et al*., 2021). In humans, 5-HTTLPR has most consistently been associated with the diagnosis or symptoms of eating disorders, the s-allele carriers having a higher vulnerability to physical or sexual abuse (Rozenblat *et al*., 2017). This cited meta-analysis included data of the ECPBHS that had reported overall similar findings (Akkermann *et al*., 2012). Adverse life events did not increase the drive for thinness and bulimia in the 5-HTTLPR l/l-homozygous participants, but such an increase was observed in the s-allele carriers, with a larger increase in subjects who had experienced more adversities. The single most impactful life event in this regard was sexual abuse. Interestingly, the s-allele carriers with no reported adverse life events had the lowest symptom scores of all groups, supporting the higher plasticity in the 5-HTTLPR s-allele carriers, also observable in the bidirectional impact of parenting styles on decision making under risk (Rehn *et al*., 2025).

Life events can thus have a different impact, and the meaning of each type of life event may vary individually owing to genetic and developmental neural factors, and learning. Therefore, offering a list of events and forming a score may be the safest while not always the optimal method for G×E. A more instructive inquiry into G×E might arise from the comparison of birth cohorts. Subjects in a birth cohort are exposed to a more similar total environment as compared to others, especially the social environment (Wedow *et al*., 2018; Virtanen *et al*., 2019), and this has consequences to e.g., psychological distress (Keyes *et al*., 2014), depressive symptoms (Yoo, 2023), drinking alcohol (Pabst *et al*., 2010; Radaev & Roshchina, 2019), and overweight and obesity (Allman-Farinelli *et al*., 2008; Opazo Breton & Gray, 2023). Studies on G×E interactions in terms of allelic variants and birth cohorts are, however, limited. With regard to drinking alcohol, the longitudinal Older Finnish Twin Cohort study detected variation by birth cohort of the contribution of genetic and environmental factors on alcohol consumption in the Finnish population (Virtanen *et al*., 2019). The ECPBHS birth cohorts were found to differ in their alcohol use debute: as expected, the younger cohort started to experiment younger, and the gender gap diminished, which are results expected on the grounds of research in other countries (Keyes *et al*., 2011). The most interesting finding, however, was the three-way interaction between birth cohort, gender and the 5-HTTLPR genotype (Vaht *et al*., 2014). The most prominent difference emerged in the comparison of s/s-homozygous females of the two cohorts: While in the older cohort this group was the last to taste alcohol, it was the earliest in the younger cohort, even surpassing boys. The age gap between these two groups was on average three years, which is sufficiently large to not be trivial: It can make a difference, if one starts drinking not in the 9th grade, but in the 6th.

But could social desirability itself, and its underlying neurobiology, be subject to birth cohort effect? Agreeableness, the social adjustment-oriented Big Five personality dimension, has been found to be sensitive to birth cohort effects, and uniquely even changeable in a cohort-dependent manner (Brandt *et al*., 2022). Agreeableness correlates negatively with the dimension ANGER of the Affective Neuroscience Personality Scale (ANPS; Davis & Panksepp, 2011; Montag *et al*., 2021) and positively with CARE, and is indeed higher in the younger cohort of the ECPBHS than in the older (Kiive *et al*., 2024a). Interestingly, Agreeableness associated in a complex manner with anxiety-related gene variants. NPY strongly attenuates anxiety in animal experiments (Heilig & Widerlöv, 1995) including social anxiety, and controls the stress axis by attenuating excessive, anxiety-provoking stimulation of corticotropin-releasing factor type 1 receptors (Kask *et al*., 1997), yet the *NPY* variants are not known to relate to anxious traits. Neither were the body composition and metabolism related *NPY* variations rs16147 and rs5574 (see above) associated with anxious personality, but in either case, homozygous subjects for one variant strongly differed by birth cohort in Agreeableness, whereas this interaction was amplified by the 5-HTTLPR genotype (Kiive *et al*., 2024a). If the interval of the ECPBHS birth cohorts would appear small, it must be recalled that the study area was a theatre for the fastest transition amongst the Countries of Central and Eastern Europe from central planning to free market economy (Allaste & Bennett, 2013), and that major economic shifts precipitate cohort effects (Sutin *et al*., 2013). That major societal changes with individualism taking the helm from collectivism could place a heavy burden on the neurobiology that has evolved to support prosocial behaviour is conceivable. Social fear conditioning has been reported to produce the most prominent alterations in the expression of NPY and serotonin-related genes throughout the brain (Hamann *et al*., 2022), thus supporting the notion that these neurochemical systems interact in social adaptive strategies, hypothetically through their interaction in orbitofrontal cortex to support behavioural flexibility (Longo *et al*., 2018). A telling case has recently been provided in the nascent research on the role of RNA m^6^A methylation in neuropsychiatry (Malovic & Pandey, 2023). Levels of RNA m^6^A methylation and expression of METTL3, a RNA methyl transferase and FTO, a RNA demethylase, have been linked to depressive disorder and suicide risk (Roy & Dwivedi, 2025), and pharmacological activation of METTL3 has anxiolytic-like properties (Kanarik *et al*., 2025). Cai et al. (2024) have reported on the association of the *FTO* rs1421085 T-allele with higher neuroticism, and we have confirmed this finding in a longitudinal analysis of the ECPBHS personality data (Kurrikoff *et al*., 2026). Interestingly, this association was apparent in the older cohort at age 25, data having been collected during the impact of the Great Recession of 2007–2009, when the subjects of the older cohort also reported much more socio-economic difficulties (Kurrikoff *et al*., 2026). This cohort effect extends to changes in dietary intake, body composition, and glucose metabolism (Katus *et al*., 2026). These findings agree well with the conclusion that the impact of total environment on brain health and behavioural outcomes, recently termed as ‘exposome’ should be taken seriously in the study of development of brain health (Robinson *et al*., 2026).

## Why impulsivity?

Impulsivity is the most common symptom across the whole DSM-5, possibly owing to its potentially devastating impact on social adaptation. Yet it appears that the meaning of premature action is in its eventual outcome, as preference of speed over accuracy, may confer benefits. The delicate balance in the multifaceted regulation of impulse control has been searched in the serotonin system (Evenden, 1999), and in the ECPBHS, an intriguing interaction between two markers of the system was revealed. Thus, impulsivity as measured with either a computer-based discrimination task or the Barratt Impulsivity Scale correlated negatively with platelet MAO activity in the s-allele carriers of the 5-HTTLPR, while among 5-HTTLPR l/l-homozygotes this association rather took an inverted U-shape, as participants with low enzyme activity exhibited lower impulsivity (Paaver *et al*., 2007). In the visual discrimination-based computer task, participants with the l/l-genotype and high platelet MAO activity had the lowest tendency of impulsive action; interestingly, this combination of biomarkers had, in females, a synergistic effect of increasing drive for thinness, a compulsivity measure (Akkermann *et al*., 2008). Which relationship aspects of impulsivity and compulsivity take, appears to depend on the inner structure of these two constructs, sometimes possible to be considered as opposite poles of one dimension, and in other instances sharing the substrate and acting synergistically (Grant & Kim, 2014).

Where impulsivity is being taken as such a negative trait, which mechanisms maintain it in the ‘survival of the fittest’? Amongst the variety of impulsivity measures collected in the ECPBHS, the favourite has been the Adaptive and Maladaptive Impulsivity Scale (AMIS), which has drawn on the concept of functional and dysfunctional impulsivity of Dickman (1990). This concept contrasts a tendency to act with little forethought when it is optimal against the tendency for thoughtlessness and inability to plan, leading to negative consequences. It appears likely that fast decision-making and action are sufficiently important to be selected for, that its other, maladaptive side can be tolerated by evolution, given that environmental contingencies vary. In the ECPBHS sample, a functional polymorphism in the neuronal nitric oxide synthase gene (*NOS1*) provided an illuminating example of G×E in impulsivity. Deletion of this gene, responsible for the glutamate-responsive production of the gaseous neurotransmitter in the CNS (Zhang & Snyder, 1993), in mice results in reduction of frontal-cortical serotonergic neurotransmission (Chiavegatto *et al*., 2001) and impulsive-aggressive behaviour, that is, however, moderated by environmental factors and develops only if living in social isolation (Chiavegatto & Nelson, 2003). Homozygocity for short (s) alleles of the dinucleotide repeat polymorphism in the promoter region of the alternative first exon 1f of human *NOS1* (*NOS1* ex1f-VNTR) was found to be associated with impulsivity and aggressiveness in diverse samples by a variety of measures, and with hypoactivation of the anterior cingulate cortex (Reif *et al*., 2009). In the ECPBHS, the *NOS1* ex1f-VNTR genotype was confirmed to be related to all analysed impulsivity constructs, and in a G×E manner that illustrated the multifaceted role of ‘impulsivity alleles’ in behaviour, and their persistence (Reif *et al*., 2011). In brief, self-reports indicated higher adaptive impulsivity in the s-allele carriers, while behavioural tests revealed a larger number of errors; they were more prone to an increase of maladaptive impulsivity if the family relationships had been inferior, but this dysfunctional measure did not increase with adverse life events, which was the case with l-allele homozygotes. Overall, when generalising across measures and conditions, heterozygotes demonstrated the best adaptive performance.

Another telling case of heterosis in impulsivity, and related apects of mental health, was observed with the *NPSR1* Asn^107^Ile (A > T; rs324981) polymorphism. Neuropeptide S (NPS) is an evolutionarily conserved peptide with restricted localisation in the CNS, and a role in arousal and anxiety (Xu *et al*., 2004). The appearance of *NPS* and its receptor *NPSR1* in the lungfish has been suggested to underlie anxiety regulation in the water-to-land transition (Wang *et al*., 2021). The rs324981 polymorphism in the human gene encoding the NPS receptor leads to exchange of an amino acid and results in a more effective signal mediation with the minor allele (Reinscheid *et al*., 2005). This corresponds to the higher prevalence of panic disorder in females and elevated anxiety sensitivity (Domschke *et al*., 2011). Responsiveness of the basolateral amygdala to fear-relevant stimuli was increased by each *NPSR1* Asn^107^Ile T-allele (Dannlowski *et al*., 2011); also suggestive of higher arousability, the T/T-homozygous subjects had less sleep (Spada *et al*., 2014), that is compatible with the role of NPS to promote wakefulness through histamin- and orexinergic neurons in animal experiments (Zhao *et al*., 2012). Interestingly, the T/T-homozygotes had higher startle response to neutral stimuli, but lower startle in response to unpleasant stimuli in caffeine vs. placebo condition (Domschke *et al*., 2012). The *NPSR1* Asn^107^Ile allele homozygous participants had highly distinct gender-dependent developmental profiles of impulsivity and mental health-related symptoms in the ECPBHS. Thus, homozygocity for the T-allele, previously associated with psychiatric vulnerability, was indeed associated with ADHD-related traits and, if combined with a higher score of stressful life events, with an increase of maladaptive as well as adaptive impulsivity (Laas *et al*., 2014b). The T/T-homozygous participants, however, did not appear as highly sensitive to inferior family environment. In contrast, the A/A-homozygous subjects had higher adaptive impulsivity and openness to experience but, if subjected to less favourable family relations, their adaptive impulsivity and extraversion decreased, whereas maladaptive impulsivity and neuroticism increased. The A/A homozygous females exposed to environmental adversities had affective and anxiety disorders more frequently; they also exhibited higher anxiety and depressiveness and lower self-esteem, and reported more frequently the experience of suicidal thoughts/behaviour (Laas *et al*., 2014a). Both environmental adversities and early alcohol use debut, as expected, increased the probability of alcohol use disorder, but its development included G×E by gender (Laas *et al*., 2015). Namely, alcohol use disorder was more common in female carriers of the A-allele and male carriers of the T-allele, with the tendency of homozygocity in either case being the most vulnerable. In summary, one can imagine two different *NPSR1* Asn^107^Ile-related pathways to the disorder: In females, environmental adversities together with the A-allele promoted neuroticism, followed by early alcohol use likely related to perceived social pressures, and in male T-allele carriers, through even earlier alcohol debut in association with impulsive and ADHD-like traits. Of note, in the population-representative ECPBHS sample, the T-allele is not the ‘minor’ allele, but the allele frequency is equal. Addictions can involve disturbances in multiple neurobiological networks, such as those responsible for the regulation of hedonic values, associative learning, formation of habits, stress response, attribution of salience, physiological withdrawal response, changes of mood and impulsive decision-making (Baler & Volkow, 2006). Individuals may vary a great deal in the relative importance of these networks in their addiction. Hence the large number of genes involved in substance use disorders, and their potential to contribute along more than one pathway.

## Aggressiveness, agonistic and antisocial behaviour

A prominent cross-diagnostic trait in psychiatry is impulsivity-related aggressiveness (Vuijk *et al*., 2020). In modern society, inter-personal aggression is considered unacceptable, and it is disruptive and detrimental to families and the whole communities (Dai & Lin, 2025). Aggressiveness is, however, a prevalent aspect of innate social behaviour across the animal kingdom, and can also be interpreted as an important component in the coordinated adaptive response to challenges presented by the environment (van Kampen, 2015). But, again both in mice and in men, aggression is increased by social deprivation in early age, and can be mitigated by stable care and reintegration (Calado *et al*., 2025).

The ECPBHS database was mined with machine-learning techniques to reveal predictors of antisocial behaviour leading to police contact in adulthood, including more than 554 behavioural and environmental measures in the hypothesis-free analysis. As expected, antisocial behaviour was more common in males; apart from this, past substance use disorder, aggressive mode of action upon provocation, and teacher-rated concentration difficulties and physical fighting in school at age 15 years appeared as the strongest risk factors (Schoenmacker *et al*., 2020). At first glance, such an outcome is not surprising, but interestingly, stress-related measures were not predictive of antisocial behaviour, unless accompanied by substance abuse. Thus, exposure to stress does not, in most instances, derail socially well-adjusted development, but sometimes it does. Among the neurobiological vulnerabilities that could predict the failure to adapt without outright aggression, the role of the suboptimal serotonergic system has received the most attention, owing to its well-documented role in impulsive acts (Coccaro *et al*., 2015).

The impact of early age serotonin levels on the development of CNS and the consequent adult behavioural integrity is, in terms of functional long-term outcome, of inverted *U*-shape, both low and excessive levels of serotonin leading to suboptimal performance (Shah *et al*., 2018). The enzyme responsible for the synthesis of serotonin in the brain is tryptophan hydroxylase 2 (TPH2). The *TPH2* gene has a promoter polymorphism −703G/T (rs4570625), the minor T-allele associated with reduced serotonin levels, and with a larger mean connectivity in the ‘rich club’, the heavily interconnected hubs (Markett *et al*., 2017). Commonly all T-allele carriers of *TPH2* −703G/T are kept together in data analysis, but in the ECPBHS it was the small (<5%) group of T/T homozygous participants that stood out with regard to their remarkably low aggressiveness and maladaptive impulsivity, and with almost total absence of anxiety disorders (Laas *et al*., 2017). This was particularly clear for male subjects, the male T/T group reporting – and retrospectively reported by their classmates – levels of aggressiveness typical for females. It is not clear yet to which degree common *TPH2* variants can reduce the availability of serotonin, but genetic modification of TPH2 levels in mice profoundly changes expression of aggressiveness, in a sex-dependent manner (Kästner *et al*., 2019).

To explore beyond serotonin, a genome-wide search of differentially methylated DNA loci was performed in peripheral blood collected from a subsample of the ECPBHS male subjects at age 15 and 25 (Pishva *et al*., 2023). The LHA total score was associated with methylation of the *PDLIM5*, a gene with a role in dendrite branching (Srivastava *et al*., 2024) and previously associated with affective disorders and schizophrenia (e.g., Kato *et al*., 2005). A subset of differentially methylated positions at age 25 were already, at age 15, predictive of later aggressiveness. Top differentially methylated positions colocalized with genetic variants previously associated, not surprisingly, with general cognitive function and educational attainment, but also with cholesterol levels. The latter finding appears highly relevant from the viewpoint of the role of low cholesterol levels in impulsive tendencies, to which a novel perspective was added in the ECPBHS (see above).

Neurotransmission by ‘classic’ neurotransmitters such as serotonin is fine-tuned at higher activity levels by a host of neuropeptides (Hökfelt *et al*., 2018), including orexins (Schöne & Burdakov, 2017). A unique study on epileptic patients monitored the release of orexin A in the amygdala and found it increased while subjects reported higher levels of anger (Blouin *et al*., 2013). Orexins, alternatively called hypocretins, are hypothalamic neuropeptides that maintain wakefulness and regulate emotional arousal as well as autonomic functions in a coordinated defence response (Johnson *et al*., 2012; Kuwaki, 2021). Orexin-expressing neurons are activated by a large variety of stressors, and hence it has been proposed that orexin neurotransmission becomes salient during highly arousing aversive conditions (Berridge *et al*., 2010). A variant of the *HCRTR1* gene (rs2271933, G1222A) in exon 7 leads to amino acid substitution (Ile^408^Val) (Meerabux *et al*., 2005). In both younger and older cohorts of the ECPBHS, the *HCRTR1* Ile^408^Val genotype was significantly associated with scores of the BPAQ and the LHA interview, the A/A homozygous participants reporting higher aggression (Harro *et al*., 2019). This was the first demonstration of the involvement of the orexin system in aggressiveness, whereas further study on the genotype revealed a variable relationship of aggressiveness with reward sensitivity (Pulver *et al*., 2020b; see below), illuminating genotype-based differences in the psychological background of aggression.

## Components of reward sensitivity?

Reward sensitivity is emerging as a major transdiagnostic component of psychiatric disorders (Oka *et al*., 2025). Reward sensitivity is a reflection of the activity in the behavioural approach system, one of the three basic motivational systems (Gray & McNaughton, 2000), that has been applied to make predictions for depression (Hazell, 2021), attention deficit-hyperactivity disorder (ADHD; Brooker *et al*., 2018), substance use (Liu & Filbey, 2024), psychopathy (De Pascalis *et al*., 2019), aggressiveness (Parker *et al*., 2022), antisocial behaviour (Hoppenbrouwers *et al*., 2015), and depressive/hypomanic symptoms in daily life (Sperry *et al*., 2025). Multiple questionnaires and computer tasks can assess reward sensitivity, and the relative inconsistency in linking reward sensitivity with several mental health conditions may derive from this multiplicity, but also from reward sensitivity being a multidimensional construct (Corr, 2016). Using the pool of item data collected in the ECPBHS, a novel scale was developed that distinguished two independently variable components of reward sensitivity, one reflecting the striving towards a multiplicity of rewards (Openness to Rewards), and the other, an excessive fixation on a particular reward (Insatiability by Reward). These two components of reward sensitivity had highly divergent relationships with personality traits in the Affective Neuroscience taxonomy: SEEKING and PLAY (and, to a lesser extent, CARE) co-varied with Openness to Rewards, while FEAR, SADNESS, and ANGER were, less strongly related to Insatiability to Rewards (Pulver *et al*., 2020a). Insatiability by Reward, but not Openness to Rewards, was linked to symptoms of ADHD and aggressiveness. The dissociation between these two facets of reward sensitivity extended to metabolic measures: Insatiability by Reward was related to body mass index, sum of skinfolds, and serum triglyceride levels, but negatively with body mass adjusted energy intake and cardiorespiratory fitness (Kiive *et al*., 2025). Openness to Rewards was, again, not related to these measures, but positively associated with physical activity and negatively with blood pressure and serum levels of glucose, insulin and cholesterol levels.

Structuring reward sensitivity this way may explain the variable findings in the literature, but of higher salience is the derived hypothesis that individual differences in reward sensitivity are part of a complex physiological variability, including energy expenditure profiles, and behavioural solutions to these inherent differences in metabolism. Reward sensitivity is evolutionarily conserved: many mammalian species show reward-related behavioural patterns similar to humans (Spear, 2011) and share its neurobiological substrate (Panksepp, 1998). It is plausible that variability within species of energy use aids survival in changeable environments and facilitates habitation of environments with different demands. This in turn has the potential to interfere with inter-individual variability of multiple other survival-related traits, not the least aggressive or agonistic behaviour.

The above-mentioned case of association of aggressiveness with the *HCRTR1* Ile^408^Val genotype (Harro *et al*., 2019) provides a tool for probing the complex relationship between reward sensitivity and aggression. The orexinergic system has been proposed to serve as a link between physical activity, metabolism, and cognition, integrating energy balance and behavioural responses (Nigro *et al*., 2025), and possibly also moderating aggressiveness. Not surprisingly, in the whole sample, aggressiveness was in positive correlation with Insatiability by Reward and not with Openness to Rewards. Intriguingly, the more aggressive genotype, the *HCRTR1* Ile^408^Val A/A homozygous group that represents 15% of the sample, had a different pattern of association (Pulver *et al*., 2020b). Namely, in the A/A homozygous subjects, the correlation between aggressiveness and Insatiability by Reward was substantially lower than in the G-allele carriers (*r* = 0.27 vs. 0.53), while the correlation between aggressiveness and Openness to Rewards, virtually zero in the G-allele carriers, was small but statistically significant in the A/A group. Thus, the higher aggressiveness of the *HCRTR1* Ile^408^Val A/A homozygous participants may relate to a distinct reward sensitivity profile. Speculatively, the A/A subjects may be more likely to get into conflict-provoking situations owing to their pursuit of multiple rewards. Such individual differences in psychological traits underlying aggression may guide improvement of individualised treatments (Blair *et al*., 2025).

## Coping strategies

Searching only for highly+specific statistically+significant differences or associations in group comparisons may obscure variability within the groups: If, say, low serotonergic capacity can predict the risk of impulsive behaviours and the consequent failure of social adjustment, then what are the resources of many subjects with genetically or developmentally low serotonergic capacity who thrive despite a presence of the measurable risk factor(s)? Such resources may range from interactions in the brain at the molecular level to action patterns at the level of community or society. The direction of causality, however, mostly remains unexplained. For example, serotonin release capacity is lower in people who reside in census tracts of lower socio-economic status (Manuck *et al*., 2005), but at present, how this difference develops begs further inquiry.

The discovery of a rare point mutation in the gene encoding the MAO-A isoenzyme that rendered the enzyme functionally ineffective and caused the development of weak impulse control and predisposition to violent outbursts in response to unexpected stressful stimuli (Brunner *et al*., 1993) led to *MAOA* often being labelled as a “gene for aggression”. Subsequently, an upstream 30 base pair variable number of tandem repeats (uVNTR) polymorphism in the promoter region of the MAO-A gene was described (Sabol et al.,^1998^), and it was found that the alleles leading to low activity MAO-A (*MAOA*-L) in males predict diminished responses of prefrontal cortex to angry and fearful faces, whereas responses of the amygdala are increased (Meyer-Lindenberg *et al*., 2006). In males, the *MAOA*-L genotype has been associated with higher aggressiveness or more violent behaviour, but not consistently with traits of common personality models (Harro & Oreland, 2016). Importantly, male *MAOA*-L subjects are more likely to present an aggressive/antisocial profile if having been maltreated in childhood (Caspi *et al*., 2002; Byrd & Manuck, 2014). No association of the *MAOA* genotype with aggressiveness was noted in the ECPBHS sample. However, consist with the concept of ‘plasticity genes’ in children from supportive environments, the *MAOA*-L genotype is protective against delinquency (Nilsson *et al*., 2014). Serious maltreatment is not common in the ECPBHS sample. Instead, we revealed significantly higher frequency of university education among the *MAOA*-L males (Kiive *et al*., 2014), and it may be relevant that the study is conducted in a traditionally highly educated region. The experiment by Frydman *et al*. (2011) that addressed financial decision-making under pressure revealed that students with the *MAOA*-L genotype did make riskier, but also more successful choices. Again, it may be relevant that the present subjects seem to have represent a socioeconomically and intellectually advantaged background.

At variance with MAO-B, regulation of MAO-A activity appears as highly prone to epigenetic control by G×E (Naoi *et al*., 2025). High perceived parental care could mitigate the increase in impulsivity by stress in female *MAOA*-L allele carriers (Kinnally *et al*., 2009), and it is conceivable that its effect was epigenetically mediated, as *MAOA* methylation is sensitive to psychotherapy (Schiele *et al*., 2018). In the ECPBHS males, methylation at an *MAOA* locus that was associated with antisocial behaviour correlated with impulsivity in an age, stress and *MAOA* genotype related manner. Thus, the correlation between the adaptive aspect of impulsivity and *MAOA* methylation in *MAOA*-L males from adverse home environments increased from 15 to 18 to 25 years of age (Kanarik *et al*., 2024). In contrast, maladaptive impulsivity had at age 18 a positive correlation of *MAOA*-L genotype with inferior family relationships and a negative correlation of *MAOA*-H with superior home environment, while both of these associations disappeared by age 25. Altogether these dynamics of *MAOA* methylation spur an hypothesis that it may serve as a marker for adaptive developmental neuroplasticity in the *MAOA*-L genotype, prone to aggression in less favourable environments.

Another functional genetic polymorphism is responsible for synaptic dopamine: in the gene encoding catechol-O-methyl transferase (*COMT*), an SNP results in the Val^158^Met (rs4680) variation, resulting in higher enzymatic activity and correspondingly lower dopamine levels with each Val-allele (Chen *et al*., 2004). Activation by unpleasant stimuli in the prefrontal cortex, amygdala and dorsal hippocampus increased with each Met-allele (Smolka *et al*., 2005), but the *COMT* Val^158^Met alleles have opposite effects in neural activation during executive and emotional tasks (Mier *et al*., 2010). Studies on global brain functional connectivity have yielded heterogeneous outcomes, wherein association of the Val-allele trends with greater resting-state connectivity and the Met-allele with greater task-based connectivity (Morris *et al*., 2020). Attempts to link the *COMT* rs4680 to distinct psychopathologies or traits have yielded some statistically significant findings, but again, overall the associations are weak, dependent perhaps on the environments of a particular investigation, and possibly on the coping strategies available there. The *COMT* genotype may relate to the preferable strategy, if this hypothesis is correct (Morris *et al*., 2020): that the Val-allele is associated with improved cognitive flexibility allowing integration of novel relevant stimuli, and the Met-allele allows improved sustained attention and targeted neural processing, particularly between limbic regions and prefrontal cortex. In the ECPBHS, either allele was related to psychiatric conditions by age 25: the Met-allele homozygous participants had a higher occurrence of alcohol use and substance use disorders while Met-allele carriers had a significantly higher occurrence of agoraphobia; the occurrence of panic disorder was higher in female Met-allele carriers than in Val/Val homozygote females (Kiive *et al*., 2024b). In contrast, the occurrence of generalised anxiety disorder was higher in Val/Val females when compared to Met-allele carriers, and Val/Val homozygous females had a higher occurrence of eating disorders than the Met-allele carriers. While interesting, this dissociation of vulnerability by the *COMT* genotype remains to be explained, whereas two other associations point at genotype-dependent coping strategies. *COMT* Val^158^Met heterozygocity, presumably representing the optimal prefrontal dopaminergic capacity, was associated in a gender-dependent manner with university education, heterozygous females being the most and heterozygous males the least educated group (Lehto *et al*., 2013). This finding was similar in the parents of the ECPBHS target subjects (Kurrikoff *et al*., 2018). Quite consistently with the ‘warrior-worrier-hypothesis’ (Stein *et al*., 2006), *COMT* Val^158^Met Val/Val homozygotes were overrepresented among enterprising occupations and the Val-allele carriers among self-classified managers, while several factors associated with this type of career selection were different in males and females (Kurrikoff *et al*., 2018).

## A commentary of sex and gender

Evolutionary models for sex differences in disease risk have posited that alleles conferring higher risk in one sex may be protective in the other, and several such polymorphisms have been identified (Harper *et al*., 2021). The collective findings in the selection of ECPBHS reports presented above, and others in the full bibliography (https://www.tai.ee/et/teadustoo/eliktu-artiklid), suggest a vast difference between males and females in G×E. This is likely to derive from differences in their respective biopsychosocial developmental curves. The highly representative sample, with minimal gender-related selection bias, may have helped reveal the contrasts. It may also be relevant that Estonia has the largest gender gaps in Europe with regard to education level (in favour of females; OECD, 2025) and income (in favour of males; Boll & Lagemann, 2018).

Some of the gender differences in G×E may result from exposure. For example, higher aggressiveness of the A/A homozygous subjects of the *HCRTR1* Ile^408^Val genotype was observed in females with higher experience of stressful life events, while in males, the A/A group was more aggressive irrespective of life event scores (Harro *et al*., 2019). Yet, male A/A subjects who become aggressive may be more sensitive to life events that they do not report, or remain out of the scope of the traditional wording of questionnaires.

A consistent finding in studies that have measured platelet MAO activity is the higher average enzyme activity in females. This ∼10% difference has been confirmed in the first pertinent study on birth cohort representative samples, the ECPBHS (Harro *et al*., 2001), while the distribution curves in males and females largely overlap (Harro & Sakala, 2025). Low platelet MAO activity has been associated with risky and law-breaking behaviours only in males, even though the meta-analysis of the association of low platelet MAO activity with the Zuckerman construct of sensation seeking revealed similar effect size for males and females (Winfield *et al*., 2025). Seriousness of the breach of law may confound gender comparisons (Oreland *et al*., 2007), and quantification of expression of antisocial behaviour can reveal low platelet MAO activity in females (Sakala *et al*., 2023). Thus, regression analysis with antisocial behaviour at two levels of frequency plus consideration of self-reported use of illicit drugs (that for most individuals remains unknown to police) either in a single occasion or repeatedly, demonstrated ‘dose-dependency’ in the relationship of antisocial behaviour and platelet MAO activity. Females, although few, with repeated police contacts had low platelet MAO activity, and even lower if multiple police contacts occurred together with repeated illicit drug use. The serotonin system in females may be more resilient to early stressors, but potentially vulnerable to additional psychosocial adversities at later stages such as adolescence. For example, specifically in female adolescents, the risk of alcohol-related problem behaviour was higher if platelet MAO activity in the lowest quartile was associated with unfavourable environment (Nilsson *et al*., 2008), and in the ECPBHS, low platelet MAO activity associated with high-risk traffic behaviour during adolescence specifically in girls (Eensoo *et al*., 2007; Luht *et al*., 2018). Such a notion also agrees with the association of early-age low cholesterol levels with maladaptive impulsivity specifically in males (Tomson-Johanson *et al*., 2020). Low cholesterol levels may in turn contribute to a decrease in brain serotonergic neurotransmission via altered microviscosity (Engelberg, 1992). Also, mouse studies suggest that females become atypically aggressive if the loss of serotonin is profound (Kästner *et al*., 2019).

For maladaptive impulsivity measures, girls appeared to be more sensitive to low levels of warmth in the family, while this was restricted to the 5-HTTLPR s-allele carriers (Paaver *et al*., 2008). In males, the s-allele related to higher maladaptive impulsivity regardless of family atmosphere. This finding resembles the sexually dimorphic response of the hormonal stress axis among rhesus macaques, where male s-allele carriers of the rh5-HTTLPR had a higher response to stress, but in case of females, experience of early adversity was additionally required (Barr *et al*., 2004).

Several further candidate genes had a rather gender-selective association with the phenotypes of interest even without consideration of life events, such as the link between *NOS1* ex1f-VNTR and aggressiveness (O‘Leary *et al*., 2020) in males, or CCK_B_ receptor gene (rs2941026) and anxiety-related measures (Lvovs *et al*., 2022) in females. Since development and genotype may impact the perception, processing and interpretation of environments then any genotype effect is a G×E effect. This notwithstanding, sex/gender differences appeared substantively in representative samples. Thus the road toward precision medicine should earnestly examine these differences in epidemiological factors and pathogenesis (Shi *et al*., 2024).

## The alternative sensitivity hypothesis

The notion of ‘vulnerability genes’ soon received a competitor concept of ‘plasticity genes’ (Belsky *et al*., 2009), according to which the much studied common gene variants such as the s-allele of the 5-HTTLPR would convey higher sensitivity to the environment (Delli Colli *et al*., 2022). Some support for this concept has emerged from animal studies focusing on the serotonin transporter, that reveal certain advantages of partial deficit of its function in more beneficial environments (Kästner *et al*., 2015). While it is plausible that certain functional variants of genes encoding key proteins make the person more malleable by some form of impactful stimuli, the concept appears to require refinement. Indeed, to say that some gene variants convey sensitivity to environment implicitly suggests that the other corresponding variants confer insensitivity to environment, which, in a Darwinian sense, may not be realistic for highly prevalent alleles. An alternative or rather progressing hypothesis to explain the contradictory findings would suggest the alternative alleles at a functionally important locus are responsive to different types of impacts in the same total environment. In one of the first two-generation analyses of the ECPBHS, risky behaviour in the 5-HTTLPR s’-allele carriers was sensitive to parental support while behaviour of the l’/l’ homozygoous participants was predicted by their fathers’ higher adaptive impulsivity, which is related to risky traffic behaviour, suggesting a possible mediation by role modelling (Tokko *et al*., 2024). Thus, the case of the 5-HTTLPR genotype serves as an example of variable sensitivity to different environmental factors within the family that impact on traffic behaviour in future, and more generally, of how functional variants of a gene can promote similar gross behaviours via different mediating pathways. Indeed, for the 5-HTTLPR, which allele is associated with resilience across divergent resilience definitions, depends on the age of subjects (Cahill *et al*., 2022). Whether the chronological age itself, or the role that the humans play in their community in an age-dependent manner, is of importance, deserves testing in future studies. The alternative sensitivity model also permits the role of agency. Cybernetic, self-regulatory models of complex behaviour that interpret temperament/personality traits acting together (Van Egeren, 2009) require that their mode of response to the environment included agency, an active engagement with external stimuli. Agency here is the ability to act, including the individual acting differently than predicted by gross models. Experimental studies in animals, using models such as social defeat have demonstrated that differences both in behaviour before a social conflict *as well as* during the social conflict itself can influence subsequent learned responses and brain activation patterns (Walker *et al*., 2009). Hence the gene×environment interaction models appear insufficient for precision medicine, and gene×environment×action interaction models will be required (Harro, 2010; Harro & Sakala, 2025).

## Strengths, limitations and opportunities

ECPBHS/EstChild has collected through five study waves a multidisciplinary database, including biosamples. Its strengths include high representation of the eligible cohorts, low attrition, and rigorous data collection. The recruitment procedure allows comparison of birth cohorts, born and developing during rapid socioeconomic transition. The large gender gaps unveiled in education and income – in opposite directions – in the country of study invite respective analyses and further scrutiny. Involvement of parents in the study enables two-generation models. The data of the ECPBHS are available to other researchers upon any reasonable request. As this is an ongoing longitudinal study, the data are maintained in a pseudonymised form; for external researchers, datasets are released in an anonymised format to ensure full protection of confidentiality. Access to the ECPBHS data requires submitting a formal data request, and requires valid approvals from the relevant Research Ethics Committee(s). Researchers must provide documentation confirming that their planned analyses meet ethical and data-protection regulations.

An obvious limitation here is that the longitudinal design with laboratory assessments has restricted the sample size. Pooling data of similar studies and replication testing are important for validation of the findings, but given the complexity of environmental variables, true reproducibility testing requires strict attention to the salience of critical features in ’enviromics’. The caveat that causality can not be inferred from statistical associations, even if made with longitudinal design, should also be borne in mind.

## Concluding notes

The ECPBHS/EstChild study has helped to interpret several controversial findings in biological psychiatry with its multidisciplinary deep phenotyping, consideration of brain-body connection and the developmental stages of behaviour. It has appeared that common biomarker effects can be understood in the context of adaptive strategies, that are subject to gene×environment interactions that are gender dependent. Further study of these birth cohorts will need to harness family aggregation effects in behaviour and mental health.

## Supporting information

10.1017/neu.2026.10072.sm001 Harro et al. supplementary material 1 Harro et al. supplementary material

10.1017/neu.2026.10072.sm002 Harro et al. supplementary material 2 Harro et al. supplementary material

10.1017/neu.2026.10072.sm003 Harro et al. supplementary material 3 Harro et al. supplementary material

10.1017/neu.2026.10072.sm004 Harro et al. supplementary material 4 Harro et al. supplementary material

## Supplementary material

The supplementary material for this article can be found at https://doi.org/10.1017/neu.2026.10072.
