# Supplementary material for: Insights into neuroscience from the representative birth cohort samples of a multidisciplinary longitudinal study
Source: Acta Neuropsychiatr. 2026 Apr 7;38:e33. doi: 10.1017/neu.2026.10072 (PMC13202404; doi:10.1017/neu.2026.10072)
Supplement: Harro et al. supplementary material 1 — Harro et al. supplementary material [file S0924270826100726sup001.docx]

**Supplementary Table 1.** Health, behaviour and performance assessment in the EstChild study waves in the younger and older cohorts.

| **Younger cohort** | | | | | | | **Older cohort** | | | | |
| --- | --- | --- | --- | --- | --- | --- | --- | --- | --- | --- | --- |
| **Domain** | **Measures and Materials** | **Age 9** | **Age 15** | **Age 18** | **Age 25** | **Age 33** | **Age 15** | **Age 18** | **Age 25** | **Age 33** |  |
| **Physiological and anthropo-metric parameters** | Blood pressure | Automatic monitor Dinamap Compact, (Johnson & Johnson Medical Ltd. Berkshire, UK) five times at 2-min intervals  n=583 | Automatic monitor Dinamap Compact, (Johnson & Johnson Medical Ltd. Berkshire, UK) five times at 2-min intervals  n=483 | Automatic monitor Dinamap Compact, (Johnson & Johnson Medical Ltd. Berkshire, UK) five times at 2-min intervals  n=453 | Automatic monitor Dinamap Compact, (Johnson & Johnson Medical Ltd. Berkshire, UK) five times at 2-min intervals  n=436 | Automatic monitor Dinamap Compact, (Johnson & Johnson Medical Ltd. Berkshire, UK) five times at 2-min intervals  n=413 | Automatic monitor Dinamap Compact, (Johnson & Johnson Medical Ltd. Berkshire, UK) five times at 2-min intervals  n=593 | Automatic monitor Dinamap Compact, (Johnson & Johnson Medical Ltd. Berkshire, UK) five times at 2-min intervals  n=436 | Automatic monitor Dinamap Compact, (Johnson & Johnson Medical Ltd. Berkshire, UK) five times at 2-min intervals  n=514 | Automatic monitor Dinamap Compact, (Johnson & Johnson Medical Ltd. Berkshire, UK) five times at 2-min intervals  n=494 |  |
|  | Height | Stadiometer Tanita HR 001 (TANITA Europe B.V., Amsterdam, Netherlands)  n=583 | Stadiometer Tanita HR 001 (TANITA Europe B.V., Amsterdam, Netherlands)  n=483 | Stadiometer Tanita HR 001 (TANITA Europe B.V., Amsterdam, Netherlands)  n=453 | Stadiometer Tanita HR 001 (TANITA Europe B.V., Amsterdam, Netherlands)  n=436 | Stadiometer Tanita HR 001 (TANITA Europe B.V., Amsterdam, Netherlands)  n=413 | Stadiometer Tanita HR 001 (TANITA Europe B.V., Amsterdam, Netherlands)  n=593 | Stadiometer Tanita HR 001 (TANITA Europe B.V., Amsterdam, Netherlands)  n=439 | Stadiometer Tanita HR 001 (TANITA Europe B.V., Amsterdam, Netherlands)  n=514 | Stadiometer Tanita HR 001 (TANITA Europe B.V., Amsterdam, Netherlands)  n=494 |  |
|  | Body mass | Calibrated beam balance  n=583 | Electronic scale (standard medical type)  n=483 | Electronic scale (standard medical type)  n=453 | Electronic scale (TANITA Europe B.V. Tanita body composition analyser BC-420MA, Amsterdam, Netherlands)  n=436 | Electronic scale (TANITA Europe B.V. Tanita body composition analyser (BC-420MA), Amsterdam, Netherlands)  n=413 | Calibrated beam balance  n=593 | Electronic scale (standard medical type)  n=439 | Electronic scale (TANITA Europe B.V. Tanita body composition analyser (BC-420MA), Amsterdam, Netherlands)  n=513 | Electronic scale (TANITA Europe B.V. Tanita body composition analyser (BC-420MA), Amsterdam, Netherlands)  n=494 |  |
|  | Body composition | OMRON, Kyoto, Japan *  n=583 | OMRON, Kyoto, Japan *  n=483 | OMRON, Kyoto, Japan * n=453 |  |  | OMRON, Kyoto, Japan *  n=593 | OMRON, Kyoto, Japan *  n=439 |  |  |  |
|  | Waist and hip circumferences | Metal anthropo-metric tape  n=583 | Metal anthropo-metric tape  n=483 | Metal anthropo-metric tape  n=453 | Metal anthropo-metric tape n=433 | Metal anthropo-metric tape  n=411 | Metal anthropo-metric tape  n=593 | Metal anthropo-metric tape  n=438 | Metal anthropo-metric tape  n=510 | Metal anthropo-metric tape n=488 |  |
|  | Skinfolds on biceps brachii, triceps brachii, subscapular, suprailiac, and medial calf | Harpenden caliper (Baty International, Burguess Hill, U.K.)  n=583 | Harpenden caliper (Baty International, Burguess Hill, U.K.)  n=483 | Harpenden caliper (Baty International, Burguess Hill, U.K.)  n=453 | Harpenden caliper (Baty International, Burguess Hill, U.K.)  n=435 | Harpenden caliper (Baty International, Burguess Hill, U.K.)  n=411 | Harpenden caliper (Baty International, Burguess Hill, U.K.)  n=593 | Harpenden caliper (Baty International, Burguess Hill, U.K.)  n=438 | Harpenden caliper (Baty International, Burguess Hill, U.K.)  n=510 | Harpenden caliper (Baty International, Burguess Hill, U.K.)  n=492 |  |
|  | Pubertal stage | Observation (Tanner and Whitehouse 1976)  n=583 | Observation  n=483 |  |  |  | Observation (Tanner and Whitehouse 1976)  n=593 |  |  |  |  |
| **Diet** | Diet diary | Food record covering a 24-hour period**  n=583 | Food record covering a 48-hour period  n=478 | Food record covering a 48-hour period  n=450 | Food record covering a 72-hour period  n=416 | Food record covering a 72-hour period  n=404 | Food record covering a 24-hour period  n=593 | Food record covering a 48-hour period  n=432 | Food record covering a 72-hour period  n=522 | Food record covering a 72-hour period  n=488 |  |
|  | Diet interview | A face-to-face, interactive interview based on food record. Portion size estimated using pictures (Haapa et al., 1985)  n=583 | A face-to-face, interactive interview based on food record. Portion size estimated using pictures  n=478 | A face-to-face, interactive interview based on food record. Portion size estimated using pictures  n=450 | A face-to-face, interactive interview based on food record. Portion size estimated using pictures  n=416 | A face-to-face, interactive interview based on food record. Portion size estimated using pictures  n=404 | A face-to-face, interactive interview based on food record. Portion size estimated using pictures (Haapa et al., 1985)  n=593 | A face-to-face, interactive interview based on food record. Portion size estimated using pictures  n=432 | A face-to-face, interactive interview based on food record. Portion size estimated using pictures  n=522 | A face-to-face, interactive interview based on food record. Portion size estimated using pictures  n=488 |  |
|  | Nutrient and food group intake evaluation | Finnish Micro-Nutrica Nutritional Analysis software, adapted to include Estonian foods (Estonian version 2.0, developed by the Food Processing Institute at Tallinn University of Technology  n=583 | Finnish Micro-Nutrica Nutritional Analysis software, adapted to include Estonian foods (Estonian version 2.0, developed by the Food Processing Institute at Tallinn University of Technology  n=478 | Estonian NutriData food consumption database (versions 4.0–15.0)  n=450 | Estonian NutriData food consumption database (versions 4.0–15.0)  n=416 | Estonian NutriData food consumption database (versions 4.0–15.0)  n=404 | Finnish Micro-Nutrica Nutritional Analysis software, adapted to include Estonian foods (Estonian version 2.0, developed by the Food Processing Institute at Tallinn University of Technology  n=593 | Finnish Micro-Nutrica Nutritional Analysis software, adapted to include Estonian foods (Estonian version 2.0, developed by the Food Processing Institute at Tallinn University of Technology  n=432 | Estonian NutriData food consumption database (versions 4.0–15.0)  n=522 | Estonian NutriData food consumption database (versions 4.0–15.0)  n=488 |  |
|  | Consumption of caffeine-containing beverages |  |  |  | Questionnaire  n=436 | Questionnaire  n=425 |  |  |  | Questionnaire  n=496 |  |
| **Physical activity and fitness** | Accelerometry-based physical activity measurement | Caltrac, Hemokinetics Inc, WI, USA  n=554 | ActiGraph GT1M, CA, USA  n=435 | ActiGraph GT1M, CA, USA  n=377 | ActiGraph GT1M, CA, USA  n=346 | GENEActiv Original, Activinsights, UK  n=400 | Caltrac, Hemokinetics Inc, WI, USA  n=563 | ActiGraph GT1M, CA, USA  n=416 | ActiGraph GT1M, CA, USA  n=442 | ActiGraph GT1M and GT3X, CA, USA  n=428 |  |
|  | Aerobic fitness test | Monark 839E, Sweden  n=580 | Monark 839E, Sweden  n=480 | Monark 839E, Sweden  n=435 | Tunturi 8, Finland  n=404 | Tunturi 8, Finland  Ergoselect 100, Germany  n=389 | Monark 839E, Sweden  n=592 | Monark 839E, Sweden  n=396 | Tunturi 8, Finland  n=480 | Tunturi 8, Finland  n=447 |  |
|  | Physical activity and exercising habits | Questionnaire  n=583 | Questionnaire  n=470 | Questionnaire  n=436 | Questionnaire  n=435 | Questionnaire  n=425 | Questionnaire  n=593 | Questionnaire  n=441 | Questionnaire  n=541 | Questionnaire  n=498 |  |
| **Health and disease** | Health self-rating, incidence of diseases | Questionnaire  n=583 | Questionnaire  n=475 | Questionnaire  n=450 | Questionnaire  n=435 | Questionnaire  n=427 | Questionnaire  n=590 | Questionnaire  n=442 | Questionnaire  n=538 | Questionnaire  n=500 |  |
| **Computer-based tests** | Cognitive skills |  | Visual Comparison Test (Dickman and Meyer, 1988)  n=378 | Stop Signal Task (SST; Logan et al., 1997) n=401  Wisconsin Card Sorting Test (WCST; Grant and Berg, 1948)  n=434 | CogShift test (Ravizza and Ciranni 2002)  n=380 | SST  n=316 |  |  | 2-back working-memory updating task (Tamm et al., 2021)  n=469 | Visual discrimination task (Forster and Lavie, 2015)  n=452 |  |
| **Biological samples collected** |  | Serum and plasma  Platelet-rich plasma  DNA  n=571 | Serum and plasma  Platelet-rich plasma  DNA  n=474 | Serum and plasma  Platelet-rich plasma  DNA  n=448 | Serum and plasma  Platelet-rich plasma  Peripheral blood mononuclear cells  DNA  n=425 | Serum and plasma  Platelet-rich plasma  Peripheral blood mononuclear cells  DNA  RNA  n=404 | Serum and plasma  Platelet-rich plasma  DNA  n=593 | Serum and plasma  Platelet-rich plasma  DNA  n=426 | Serum and plasma  Platelet-rich plasma  DNA  n=506 | Serum and plasma  Platelet-rich plasma  Peripheral blood mononuclear cells  DNA  RNA  n=492 |  |
| **Biological analyses already conducted** | Clinical biochemistry | Triglycerides, cholesterol, HDL-cholesterol, LDL-cholesterol, glucose, insulin, homocysteine  n=571 | Triglycerides, cholesterol, HDL-cholesterol, LDL-cholesterol, glucose, insulin, C-reactive protein, homocysteine  n=474 | Triglycerides, cholesterol, HDL-cholesterol, LDL-cholesterol, glucose, insulin, C-reactive protein, homocysteine  n=448 | Triglycerides, cholesterol, HDL-cholesterol, LDL-cholesterol, glucose, insulin, C-reactive protein  n=425 | Triglycerides, cholesterol, HDL-cholesterol, LDL-cholesterol, glucose, insulin, C-reactive protein, homocysteine  n=404 | Triglycerides, cholesterol, HDL-cholesterol, LDL-cholesterol, glucose, insulin, homocysteine  n=593 | Triglycerides, cholesterol, HDL-cholesterol, LDL-cholesterol, glucose, insulin, C-reactive protein, homocysteine  n=426 | Triglycerides, cholesterol, HDL-cholesterol, LDL-cholesterol, glucose, insulin, C-reactive protein, homocysteine  n=506 | Triglycerides, cholesterol, HDL-cholesterol, LDL-cholesterol, glucose, insulin, C-reactive protein, homocysteine  n=492 |  |
|  | Genetics | Candidate genes (SNPs and VNTRs) n=1235  GWAS (Infinitum Global Screening Array-24+v2.0) n=1235 | | | | | | | | |  |
|  | Epigenetics  (n variable) | DNA methylation (selected loci) | DNA methylation (selected loci)  EWAS (Infinum Methylation-EPIC BeadChip)*** | DNA methylation (selected loci) | DNA methylation (selected loci)  EWAS (Infinum Methylation-EPIC BeadChip)*** | DNA methylation (selected loci) | DNA methylation (selected loci) | DNA methylation (selected loci) | DNA methylation (selected loci) | DNA methylation (selected loci) |  |
|  | Platelet MAO activity | Platelet MAO activity  n=544 | Platelet MAO activity  n=471 | Platelet MAO activity  n=424 | Platelet MAO activity  n=412 | Platelet MAO activity  n=383 | Platelet MAO activity  n=581 | Platelet MAO activity  n=415 | Platelet MAO activity  n=498 | **** |  |

* OMRON device was used only for body fat percentage as feedback, not for scientific analysis

** Parental assistance

*** Preselected subjects

**** Samples lost in a freezer emergency

References are given only at the earliest use of the instrument in either cohort.

**References**

Dickman SJ and Meyer DE (1988) Impulsivity and speed-accuracy tradeoffs in information processing. *Journal of Personality and Social Psychology* **54**, 274–290. doi: 10.1037/0022-3514.54.2.274

Forster S and Lavie N (2015) Establishing the attention-distractibility trait. *Psychological Science* **27**, 203-212.doi: 10.1177/0956797615617761

Grant DA and Berg EA (1948) Wisconsin Card Sorting Test [Database record]. APA PsycTests. doi: 10.1037/t31298-000

Haapa E, Toponen T, Pietinen P and Räsänen L (1985) Annoskuvakirja. Helsingi: Kansanterveyslaitas

Logan GD, Schachar RJ and Tannock R (1997) Impulsivity and inhibitory control. *Psychological Science* **8**, 60-64. doi: 10.1111/j.1467-9280.1997.tb00545.x

Ravizza SM and Ciranni MA (2002) Contributions of the prefrontal cortex and basal ganglia to set shifting. *Journal of Cognitive Neuroscience* **14**, 472-483. doi: 10.1162/089892902317361985

Tamm G, Kreegipuu K and Harro J (2021) Platelet MAO activity and *COMT* Val^158^Met genotype interaction predicts visual working memory updating efficiency. *Behavioural Brain Research* **407**, 113255. doi: 10.1016/j.bbr.2021.113255

Tanner JM and Whitehouse RH (1976) Clinical longitudinal standards for height, weight, height velocity, weight velocity, and stages of puberty. *Archives of Disease in Childhood* **51**, 170-179. doi: 10.1136/adc.51.3.170
