# Supplementary material for: Insights into neuroscience from the representative birth cohort samples of a multidisciplinary longitudinal study
Source: Acta Neuropsychiatr. 2026 Apr 7;38:e33. doi: 10.1017/neu.2026.10072 (PMC13202404; doi:10.1017/neu.2026.10072)
Supplement: Harro et al. supplementary material 3 — Harro et al. supplementary material [file S0924270826100726sup003.docx]

**Supplementary Table 2.** Questionnaires and structured interviews used in the Estonian Children Personality Behaviour and Health Study by birth cohort and study wave.

| **Younger cohort** | | | | | | | **Older cohort** | | | | |
| --- | --- | --- | --- | --- | --- | --- | --- | --- | --- | --- | --- |
| **Domain** | **Measures** | **Age 9** | **Age 15** | **Age 18** | **Age 25** | **Age 33** | **Age 15** | **Age 18** | **Age 25** | **Age 33** |  |
| **Personality** | Five factor personality traits | EBBFI (Laidra et al., 2006) parent-rated, teacher-rated | EBBFI, parent-rated  EE.PIP-NEO (Mõttus et al., 2006) | EBBFI, parent-rated  S5 (Konstabel et al., 2012) self-rated, parent-rated | EE.PIP-NEO | EE.PIP-NEO | EBBFI (Laidra et al., 2006) parent-rated, self-rated,  teacher-rated | EBBFI, parent-rated, teacher-rated  NEO-PI-R (Kallasmaa et al., 2000) | EE.PIP-NEO (Mõttus et al., 2006) | EE.PIP-NEO |  |
|  | Affective Neuroscience Personality |  |  |  | ANPS (Davis et al., 2003; Harro et al., 2019) | ANPS |  |  |  | ANPS (Davis et al., 2003; Harro et al., 2019) |  |
| **Cognitive abilities** | Fluid intelligence |  | RPM, sets C,D (Raven et al.,1998; Lynn et al., 2002) |  | RPM, sets C,D,E | RPM, sets C,D,E |  |  | RPM, sets C,D (Raven et al.,1998; Lynn et al., 2002) | RPM, sets C,D,E |  |
| **Psychological traits** | State and trait anxiety |  | STAI-S (Spielberger et al., 1983) | STAI-S, STAI-T | STAI-S, STAI-T | STAI-S, STAI-T |  |  | STAI-S, STAI-T  (Spielberger et al., 1983) | STAI-S, STAI-T |  |
|  | Depressiveness |  | BDI (Beck et al., 1996) | MÅDRS (Montgomery and Åsberg, 1979) | MÅDRS | MÅDRS |  | MÅDRS (Montgomery and Åsberg, 1979) | MÅDRS | MÅDRS |  |
|  | Self-esteem |  |  | RSES (Rosenberg, 1965; Pullmann and Allik, 2000) | RSES | RSES |  |  | RSES (Rosenberg, 1965; Pullmann & Allik, 2000) | RSES |  |
|  | Fears |  |  | Fears (Tulviste et al., 2015) | Fears | Fears |  |  | Fears (Tulviste et al., 2015) | Fears |  |
|  | Affect intensity |  |  | AIM (Larsen, 1984) |  |  |  |  | AIM (Larsen, 1984) |  |  |
|  | Inattention and hyperactivity | Hyperactivity scale (af Klinteberg and Oreland, 1995), teacher- and parent-rated | Hyperactivity scale, teacher-rated  SNAP-IV (Swanson, 1992) teacher- and parent-rated | Hyperactivity scale, teacher-rated  SNAP-IV, teacher- and parent-rated | ASRS (Kessler et al., 2005) | ASRS  ATTC (Derryberry and Reed, 2002)  Simple ADHD questionnaire | Hyperactivity scale (af Klinteberg and Oreland, 1995), teacher-and parent-rated | Hyperactivity scale, teacher-rated  SNAP-IV (Swanson, 1992) teacher- and parent-rated | ASRS (Kessler et al., 2005) | ASRS  ATTC (Derryberry and Reed, 2002)  Simple ADHD questionnaire |  |
|  | Impulsivity |  | AMIS (Paaver et al., 2006)  BIS-11 (Patton et al., 1995) | AMIS  BIS-11 | AMIS  BIS-11 | AMIS  BIS-11 |  | AMIS (Paaver et al., 2006) | AMIS  BIS-11 (Patton et al., 1995) | AMIS  BIS-11 |  |
|  | Reward sensitivity |  |  |  | ROIS (Pulver et al., 2020) | ROIS-2 (Pulver et al., 2020) |  |  |  | ROIS (Pulver et al., 2020) |  |
|  | Aggressiveness | Af Klinteberg Hyperactivity scale (af Klinteberg and Oreland, 1995; Kiive et al., 2010), teacher- and parent-rated | Af Klinteberg Hyperactivity scale, teacher-rated | Af Klinteberg Hyperactivity scale, teacher-rated | BPAQ (Buss and Perry, 1992)  IBS (Espelage and Holt, 2001)  APQ (O´Connor et al., 2001)  KVLS-KAKK | BPAQ (Buss and Perry, 1992) | Af Klinteberg Hyperactivity scale (af Klinteberg and Oreland, 1995; Kiive et al., 2010), teacher-and parent-rated | Af Klinteberg Hyperactivity scale, teacher-rated |  | BPAQ (Buss and Perry, 1992)  IBS (Espelage and Holt, 2001)  APQ (O´Connor et al., 2001)  KVLS-KAKK |  |
| **Behaviour and lifestyle** | Traffic behaviour |  |  | Traffic safety questionnaire (Luht et al., 2018) | Traffic safety questionnaire DBQ (Reason et al., 1990)  DAS (Deffenbacher et al., 1994) | Traffic safety questionnaire DBQ  DAS |  |  | Traffic safety questionnaire (Luht et al., 2018)  DBQ (Reason et al., 1990)  DAS (Deffenbacher et al., 1994)  DSI (Lajunen and Summala, 1995) | Traffic safety questionnaire DBQ  DAS |  |
|  | Alcohol, tobacco, illicit drug use | Questionnaire (Merenäkk et al., 2011) | Questionnaire | Questionnaire | Questionnaire | Questionnaire  AUDIT (Saunders et al., 1993)  FTND (Heatherton et al., 1991) | Questionnaire (Merenäkk et al., 2011) | Questionnaire | Questionnaire | Questionnaire  AUDIT (Saunders et al., 1993)  FTND (Heatherton et al., 1991) |  |
|  | Internet Addiction Test |  |  |  |  | IAT (Young, 1998) |  |  |  | IAT (Young, 1998) |  |
| **Relationships** | Relationships in the family | Questionnaire | TFRS (Paaver et al., 2008) | TFRS |  |  | Questionnaire | TFRS (Paaver et al., 2008)** |  |  |  |
|  | Relationships with parents |  |  | PARQ/Control (Rohner & Khaleque 2005; Tulviste et al., 2015)*** | Relationships with mother and father questionnaire | Relationships with mother and father questionnaire |  |  | PARQ/Control (Rohner & Khaleque 2005; Tulviste et al., 2015)***  Relationships with mother and father questionnaire | Relationships with mother and father questionnaire |  |
|  | Relationship with partner |  |  |  | Relationship with partner questionnaire | Relationship with partner questionnaire |  |  | Relationship with partner questionnaire | Relationship with partner questionnaire |  |
|  | Relationships in school | Questionnaire (Vaht et al., 2016) | Questionnaire | Questionnaire |  |  | Questionnaire (Vaht et al., 2016) | Questionnaire |  |  |  |
|  | Socialization values questionnaire |  |  |  | Questionnaire (Tulviste, 2013) |  |  |  | Questionnaire (Tulviste, 2013) |  |  |
| **Socio-economic situation and life events** | Socioeconomic information | Questionnaire | Questionnaire | Questionnaire | Questionnaire | Questionnaire | Questionnaire | Questionnaire | Questionnaire | Questionnaire |  |
|  | Stress and stressful life events |  | Questionnaire (Akkermann et al., 2012) | Questionnaire (Akkermann et al., 2012) | Questionnaire (Lehto et al., 2016) | Questionnaire (Lehto et al., 2016) | Questionnaire (Akkermann et al., 2012) | Questionnaire (Akkermann et al., 2012) | Questionnaire (Lehto et al., 2016) | Questionnaire (Lehto et al., 2016) |  |
|  | Work-related psychosocial factors |  |  |  | COPSOQ (Kristensen et al., 2005) | COPSOQ |  |  | COPSOQ (Kristensen et al., 2005) | COPSOQ |  |
|  | Life satisfaction |  |  |  | SLS (Diener et al., 1985) | SLS |  |  |  | SLS (Diener et al., 1985) |  |
| **Psychiatry** | Psychiatric disorders |  |  |  | MINI 5.0.0 (Sheehan et al., 1998; Shlik et al., 1999) |  |  |  | MINI 5.0.0 (Sheehan et al., 1998; Shlik et al., 1999) | MINI 5.0.0 (Sheehan et al., 1998; Shlik et al., 1999) |  |
|  | History of aggression |  |  |  | Life history of aggression (Coccaro et al., 1997) |  |  |  |  | Life history of aggression (Coccaro et al., 1997) |  |
|  | Eating behaviour |  | EDI-2 (Garner, 1991; Podar et al., 1999) | EDI-2 | EDI-2* | EDI-2* |  | EDI-2 (Garner, 1991; Podar et al., 1999) | EDI-2 | EDI-2* |  |

* Only females completed the questionnaire

** Only one subscale was used

*** Participants provided data only on mothers

Self-reports if not stated otherwise. Self-rating is only mentioned if proxy reports were also collected.

References are given only at the earliest use of the instrument in either cohort.

Abbreviations: AIM- Affect Intensity Measure; AMIS- Adaptive and Maladaptive Impulsivity Scale; ANPS- Affective Neuroscience Personality Scale; APQ- Aggressive Provocation Questionnaire; ASRS- Adult ADHD Self-Report Scale; ATTC- Attention Control Scale; AUDIT- Alcohol Use Disorders Identification Test; BDI- Beck Depression Inventory; BIS- Barratt Impulsiveness Scale; BIS/BAS- behavioural inhibition and behavioural activation system; BPAQ- Buss and Perry Aggression Questionnaire; BSSQ- Brief Social Support Questionnaire; COPSOQ- Copenhagen Psychosocial Questionnaire; DAS- Driver Anger Scale; DBQ- Driver Behaviour Questionnaire; DSI- Driver Skill Inventory; EBBFI- Estonian Brief Big Five Inventory; EDI-2- Eating Disorders Inventory-2; EE.PIP-NEO- Estonian adaptation IPIP NEO; FTND- Fagerström Test for Nicotine Dependence; IAT- Internet Addiction Test; IBS- Illinois Bully Scale; MÅDRS- Montgomery-Åsberg Depression Rating Scale; MINI 5.0.0- Mini-International Neuropsychiatric Interview; NEO-PI-R-Revised- NEO Personality Inventory; PARQ/Control- Parental Acceptance-Rejection/Control Questionnaire; ROIS- Reward Openness and Insatiability Scale; RPM- Raven Progressive Matrices; RSES- Rosenberg Self-Esteem Scale; S5- Short Five; SLS- Satisfaction with Life Scale; SNAP-IV- Swanson, Nolan and Pelham Questionnaire IV; STAI- The Spielberger State Trait Anxiety Inventory; TFRS- Tartu Family Relationships Scale

**References**

Akkermann K, Kaasik K, Kiive E, Nordquist N, Oreland L and Harro J (2012) The impact of adverse life events and the serotonin transporter gene promoter polymorphism on the development of eating disorder symptoms. *Journal of Psychiatric Research* **46**, 38-43. doi: 10.1016/j.jpsychires.2011.09.013

Beck AT, Ward CH, Mendelson M, Mock J and Erbaugh J (1961) An inventory for measuring depression. *Archives of General Psychiatry* **4***,* 561–571. doi: 10.1001/archpsyc.1961.01710120031004

Buss AH and Perry M (1992) The Aggression Questionnaire. *Journal of Personality and Social Psychology* **63**, 452–459. doi: 10.1037/0022-3514.63.3.452

Coccaro EF, Berman ME and Kavoussi RJ (1997) Assessment of life-history of aggression: Development and psychometric characteristics. *Psychiatry Research* **73**, 147–157. doi: 10.1016/S0165-1781(97)00119-4

Davis KL, Panksepp J and Normansell L (2003) The Affective Neuroscience Personality Scales: Normative data and implications. *Neuro-Psychoanalysis* **5**, 57–69. doi: 10.1080/15294145.2003.10773410

Deffenbacher JL, Oetting ER and Lynch RS (1994) Development of a Driving Anger Scale. *Psychological Reports* **74**, 83-91. doi: 10.2466/pr0.1994.74.1.83

Derryberry D and Reed MA (2002) Anxiety-related attentional biases and their regulation by attentional control. *Journal of Abnormal Psychology* **111**, 225–236. https://doi.org/10.1037//0021-843x.111.2.225

Diener E, Emmons RA, Larsen RJ and Griffin S (1985) The Satisfaction With Life Scale. *Journal of Personality Assessment* **49**, 71–75. doi: 10.1207/s15327752jpa4901_13

Espelage DL and Holt MK (2001) Bullying and victimization during early adolescence: Peer influences and psychosocial correlates. *Journal of Emotional Abuse* **2**, 123–142. doi: 10.1300/J135v02n02_08

Garner DM (1991) Eating Disorder Inventory-2 professional manual. Odessa (Fla): Psychological Assessment Resources

Harro J, Laas K, Eensoo D, Kurrikoff T, Sakala K, Vaht M, Parik J, Mäestu J and Veidebaum T (2019) Orexin/hypocretin receptor gene (*HCRTR1*) variation is associated with aggressive behaviour. *Neuropharmacology* **156**, 107527. doi: 10.1016/j.neuropharm.2019.02.009

Heatherton TF, Kozlowski LT, Frecker RC and Fagerström, K (1991) *Fagerström Test for Cigarette Dependence (FTND)*. APA PsycTests. doi: 10.1037/t03773-000

Kallasmaa T, Allik J, Realo, A and McCrae RR (2000) The Estonian version of the NEO‐PI‐R: an examination of universal and culture‐specific aspects of the Five‐Factor Model. *European Journal of Personality* **14**, 265-278. doi: 10.1002/1099-0984(200005/06)14:3<265::AID-PER376>3.0.CO;2-B

Kessler RC, Adler L, Ames M, Demler O, Faraone S, Hiripi E, Howes MJ, Jin R, Secnik K, Spencer T, Ustun TB and Walters EE (2005) The World Health Organization Adult ADHD Self-Report Scale (ASRS): a short screening scale for use in the general population. *Psychological Medicine* **35**, 245–256. https://doi.org/10.1017/s0033291704002892

Kiive E, Kurrikoff T, Mäestu J and Harro J (2010) Effect of alpha_2A_-adrenoceptor C-1291G genotype and maltreatment on hyperactivity and inattention in adolescents. *Progress in Neuro-Psychopharmacology & Biological Psychiatry* **34**, 219-224. doi: 10.1016/j.pnpbp.2009.11.011

af Klinteberg B and Oreland L (1995) Hyperactive and aggressive behaviors in childhood as related to low platelet monoamine oxidase (MAO) activity at adult age: A longitudinal study of male subjects. *Personality and Individual Differences* **19**, 373–383. doi: 10.1016/0191-8869(95)00075-H

Konstabel K, Lönnqvist J, Walkowitz G, Konstabel K and Verkasalo M (2012) The ‘Short Five’ (S5): Measuring personality traits using comprehensive single items. *European Journal of Personality* **26**, 13-29. doi: 10.1002/per.813

Kristensen TS, Hannerz H, Høgh A and Borg V (2005) The Copenhagen Psychosocial Questionnaire--a tool for the assessment and improvement of the psychosocial work environment. *Scandinavian Journal of Work, Environment & Health* **31**, 438–449. https://doi.org/10.5271/sjweh.948

Laidra K, Allik J, Harro M, Merenäkk L and Harro J (2006) Agreement among adolescents, parents, and teachers on adolescent personality. *Assessment* **13**, 187–196. doi: 10.1177/1073191106287125

Lajunen T and Summala H (1995) Driving experience, personality and skill- and safety-motive dimensions in drivers’ self-assessments. *Personality and Individual Differences* **19**, 307-318. doi: 10.1016/0191-8869(95)00068-H

Larsen RJ (1984) Theory and measurement of affect intensity as an individual difference characteristic. *Dissertation Abstracts International* 85, 2297B

Lehto K, Mäestu J, Kiive E, Veidebaum T and Harro J (2016) BDNF Val66Met genotype and neuroticism predict life stress: A longitudinal study from childhood to adulthood. *European Neuropsychopharmacology* **26**, 562–569. doi: 10.1016/j.euroneuro.2015.12.029

Luht K, Eensoo D, Tooding LM and Harro J (2018) The association of measures of the serotonin system, personality, alcohol use, and smoking with risk-taking traffic behavior in adolescents in a longitudinal study. *Nordic Journal of Psychiatry* **72**, 9-16. doi: 10.1080/08039488.2017.1368702

Lynn R, Allik J, Pullmann H and Laidra K (2002) A study of intelligence in Estonia. *Psychological Reports* **91**, 1022–1026. doi: 10.2466/pr0.2002.91.3.1022

Merenäkk L, Mäestu J, Nordquist N, Parik J, Oreland L, Loit HM and Harro J (2011) Effects of the serotonin transporter (5-HTTLPR) and α_2A_-adrenoceptor (C-1291G) genotypes on substance use in children and adolescents: a longitudinal study. *Psychopharmacology (Berl)* **215**, 13-22. doi: 10.1007/s00213-010-2109-z

Montgomery SA and Åsberg M (1979) A new depression scale designed to be sensitive to change.  *British Journal of Psychiatry* **134**, 382–389. doi: 10.1192/bjp.134.4.382

Mõttus R, Pullmann H and Allik J (2006) Toward more readable Big Five Personality Inventories. *European Journal of Psychological Assessment* **22**, 149–157. doi/10.1027/1015-5759.22.3.149

O’Connor DB, Archer J and Wu FWC (2001) Measuring aggression: Self-reports, partners, and responses to provoking scenarios. *Aggressive Behavior* 27, 79-101. doi:10.1002/ab.2

Paaver M, Eensoo D, Pulver A and Harro J (2006) Adaptive and maladaptive impulsivity, platelet monoamine oxidase (MAO) activity and risk-admitting in different types of risky drivers. *Psychopharmacology (Berl)* **186**, 32-40. doi: 10.1007/s00213-006-0325-3

Paaver M, Kurrikoff T, Nordquist N, Oreland L and Harro J (2008) The effect of 5-HTT gene promoter polymorphism on impulsivity depends on family relations in girls. *Progress in Neuro-Psychopharmacology & Biological Psychiatry* **32**, 1263-1268. doi: 10.1016/j.pnpbp.2008.03.021

Patton JH, Stanford MS and Barratt ES (1995) Factor structure of the Barratt impulsiveness scale. *Journal of Clinical Psychology* **51**, 768–774. https://doi.org/10.1002/1097-4679(199511)51:6<768::aid-jclp2270510607>3.0.co;2-1

Podar I, Hannus A and Allik J (1999) Personality and affectivity characteristics associated with eating disorders: a comparison of eating disordered, weight-preoccupied, and normal samples. *Journal of Personality Assessment* **73**, 133–147. doi: 10.1207/S15327752JPA730109

Pullmann H and Allik J (2000) The Rosenberg Self-Esteem Scale: Its dimensionality, stability and personality correlates in Estonian. *Personality and Individual Differences* **28**, 701–715. doi: 10.1016/S0191-8869(99)00132-4

Pulver A, Kiive E and Harro J (2020) Reward sensitivity, affective neuroscience personality, symptoms of attention-deficit/hyperactivity disorder, and *TPH2*-703G/T (rs4570625) genotype. *Acta Neuropsychiatrica* **32**, 247–256. doi: 10.1017/neu.2020.18

Raven J, Raven JC and Court JH (1998) Manual for Raven's Progressive Matrices and Vocabulary Scales, Section 1: General Overview. San Antonio, TX: Harcourt Assessment.

Reason J, Manstead A, Stradling S, Baxter J and Campbell K (1990) Errors and violations on the roads: a real distinction? *Ergonomics* **33**, 1315-1332. doi: 10.1080/00140139008925335

Rohner RP and Khaleque A (2005) Parental acceptance-rejection questionnaire (PARQ): Test manual. *Handbook for the study of parental acceptance and rejection* **4**, 43-106.

Rosenberg M (1965) Rosenberg self-esteem scale (RSE). *Acceptance and Commitment Therapy*. Measures Package, 61.

Saunders JB, Aasland OG, Babor TF, De La Fuente JR and Grant M (1993) Development of the Alcohol Use Disorders Identification Test (AUDIT): WHO Collaborative Project on early detection of persons with harmful alcohol consumption-II. *Addiction* **88**, 791–804. doi: 10.1111/j.1360-0443.1993.tb02093.x

Sheehan DV, Lecrubier Y, Sheehan KH, Amorim P, Janavs J, Weiller E, Hergueta T, Baker R and Dunbar GC (1998) The Mini-International Neuropsychiatric Interview (M.I.N.I.): the development and validation of a structured diagnostic psychiatric interview for DSM-IV and ICD-10.  *Journal of Clinical Psychiatry* **59** (Suppl 20), 22–57.

Shlik J, Aluoja A and Kihl E (1999) MINI 5.0.0. Mini rahvusvaheline neuropsühhiaatriline intervjuu DSM – IV. Estonian version of MINI international neuropsychiatric interview.

Spielberger CD, Gorsuch RL, Lushene PR, Vagg PR and Jacobs AG (1983) Manual for the State-Trait Anxiety Inventory. Consulting Psychologists Press, Inc., Palo Alto.

Swanson JM (1992) School-Based Assessment and Interventions for ADD Students. Irvine, CA: KC Publications.

Tulviste T (2013) Socialization values of mothers and fathers: Does the child’s age matter? *Trames* **17**, 129-140. DOI: 10.3176/tr.2013.2.02

Tulviste T, Kiive E, Akkermann K and Harro J (2015) Fears in the general population: More frequent in females and associated with the serotonin transporter promoter polymorphism and perceived relationship with mothers. *Journal of Child Neurology* **30**, 1459-1465. doi: 10.1177/0883073815570151

Vaht M, Kurrikoff T, Laas K, Veidebaum T and Harro J (2016) Oxytocin receptor gene variation rs53576 and alcohol abuse in a longitudinal population representative study. *Psychoneuroendocrinology* **74**, 333-341. doi: 10.1016/j.psyneuen.2016.09.018

Young KS (1998) Internet Addiction: The Emergence of a New Clinical Disorder. *CyberPsychology & Behavior* **1**, 237-244
