# Supplementary material for: Insights into neuroscience from the representative birth cohort samples of a multidisciplinary longitudinal study
Source: Acta Neuropsychiatr. 2026 Apr 7;38:e33. doi: 10.1017/neu.2026.10072 (PMC13202404; doi:10.1017/neu.2026.10072)
Supplement: Harro et al. supplementary material 4 — Harro et al. supplementary material [file S0924270826100726sup004.docx]

**Supplementary Table 3.** Questionnaires and measurements for parents in the Estonian Children Personality Behaviour and Health Study

| **Domain** | **Measures and materials** | **Previously collected data** | **2011-2013 Sampling of Parents** |
| --- | --- | --- | --- |
| **Personality** | Five factor personality traits | EBBFI (Laidra et al., 2006)  S5 (Konstabel et al., 2012) | EE.PIP-NEO (Mõttus et al., 2006) |
| **Cognitive abilities** | Fluid intelligence |  | RPM, sets C,D,E (Raven et al.,1998; Lynn et al., 2002) |
| **Psychological traits** | State and trait anxiety |  | STAI-S, STAI-T (Spielberger et al., 1983) |
|  | Depressiveness |  | MÅDRS (Montgomery and Åsberg, 1979) |
|  | Self-esteem |  | RSES (Rosenberg, 1965; Pullmann and Allik, 2000) |
|  | Fears |  | Fears (Tulviste et al., 2015) |
|  | Affect intensity |  | AIM (Larsen, 1984) |
|  | Inattention and hyperactivity |  | ASRS (Kessler et al., 2005) |
|  | Impulsivity |  | AMIS (Paaver et al., 2006)  BIS-11 (Patton et al., 1995) |
| **Behaviour** | Traffic behaviour | Traffic safety questionnaire (Luht et al., 2018) | Traffic safety questionnaire |
|  | Alcohol, tobacco, illicit drug use | Questionnaire (Merenäkk et al., 2011) | Questionnaire |
| **Relationships** | Relationships in the family |  | TFRS (Paaver et al., 2008) |
|  | Relationships with parents |  | PARQ/Control (Rohner and Khaleque 2005; Tulviste et al., 2015), both parents completed the questionnaire for their own parents |
|  | Relationship with partner |  | Relationship with partner questionnaire |
|  | Socialization values questionnaire | Questionnaire (Tulviste, 2013) |  |
| **Socio-economic situation and life events** | Socioeconomic information | Questionnaire | Questionnaire |
|  | Stress and stressful life events |  | Questionnaire (Lehto et al., 2016) |
|  | Work-related psychosocial factors |  | COPSOQ (Kristensen et al., 2005) |
|  | Life satisfaction |  | SLS (Diener et al., 1985) |
| **Psychiatry** | Psychiatric disorders |  | MINI 5.0.0 (Sheehan et al., 1998; Shlik et al., 1999) |
|  | Eating behaviour | Some questions about eating behaviours | EDI-2 (Garner, 1991; Podar et al., 1999), only mothers completed the questionnaire |
| **Physiological and anthropometric parameters** | Blood pressure |  | Automatic blood pressure monitor ( Dinamap Compact, Johnson & Johnson Medical Ltd. Berkshire, UK), five times at 2-min intervals |
|  | Height |  | Stadiometer ( Tanita HR 001, TANITA Europe B.V., Amsterdam, Netherlands) |
|  | Body mass |  | Electronic scale (Tanita body composition analyser (BC-420MA), TANITA Europe B.V., Amsterdam, Netherlands) |
|  | Waist and hip circumference |  | Metal anthropometric tape |
|  | Skinfolds on biceps brachii, triceps brachii, subscapular, suprailiac, and medial calf |  | Harpenden caliper (Baty International, Burguess Hill, U.K.) |
| **Health** | Health status questionnaire | Questionnaire | Questionnaire |
| **Diet** | Diet diary |  | Food record covering a 72-hour period |
|  | Diet interview |  | A face-to-face, interactive interview with validated food record data. Portion sizes estimated using pictures (Haapa et al., 1985) |
|  | Nutrient and food group intake evaluation |  | Estonian NutriData food consumption database (versions 4.0–15.0) |
| **Physical performance** | Accelerometry-based physical activity measurement |  | ActiGraph, CA, USA |
|  | Physical activity and exercise information | Questionnaire | Questionnaire |
| **Computer-based tests** | Cognitive skills |  | Stop Signal Task (SST; Logan et al., 1997) |
| **Biological samples** | Plasma and serum |  | Cardiometabolic biomarkers |
|  | DNA |  | Genotyping of functional polymorphisms |
|  | Platelet-rich plasma |  | Platelet MAO activity |

Abbreviations: AIM- Affect Intensity Measure; AMIS- Adaptive and Maladaptive Impulsivity Scale; ASRS- Adult ADHD Self-Report Scale; BIS- Barratt Impulsiveness Scale; COPSOQ- Copenhagen Psychosocial Questionnaire; EBBFI- Estonian Brief Big Five Inventory; EDI-2- Eating Disorders Inventory-2; EE.PIP-NEO- Estonian adaptation IPIP NEO; MÅDRS- Montgomery-Åsberg Depression Rating Scale; MINI 5.0.0- Mini-International Neuropsychiatric Interview; PARQ/Control- Parental Acceptance-Rejection/Control Questionnaire; RPM- Raven Progressive Matrices; RSES- Rosenberg Self-Esteem Scale; S5- Short Five; SLS-Satisfaction with Life Scale; STAI- The Spielberger State Trait Anxiety Inventory; TFRS- Tartu Family Relationships Scale

References are given only at the earliest use of the instrument.

**References**

Diener E, Emmons RA, Larsen RJ and Griffin S (1985) The Satisfaction With Life Scale. *Journal of Personality Assessment* **49**, 71–75. doi: 10.1207/s15327752jpa4901_13

Garner DM (1991) Eating Disorder Inventory-2 professional manual. Odessa (Fla): Psychological Assessment Resources

Haapa E, Toponen T, Pietinen P and Räsänen L (1985) Annoskuvakirja. Helsingi: Kansanterveyslaitas

Kessler RC, Adler L, Ames M, Demler O, Faraone S, Hiripi E, Howes MJ, Jin R, Secnik K, Spencer T, Ustun TB and Walters EE (2005) The World Health Organization Adult ADHD Self-Report Scale (ASRS): a short screening scale for use in the general population. *Psychological Medicine* **35**, 245–256. https://doi.org/10.1017/s0033291704002892

Konstabel K, Lönnqvist J, Walkowitz G, Konstabel K and Verkasalo M (2012) The ‘Short Five’ (S5): Measuring personality traits using comprehensive single items. *European Journal of Personality* **26**, 13-29. doi: 10.1002/per.813

Kristensen TS, Hannerz H, Høgh A and Borg V (2005) The Copenhagen Psychosocial Questionnaire--a tool for the assessment and improvement of the psychosocial work environment. *Scandinavian Journal of Work, Environment & Health* **31**, 438–449. https://doi.org/10.5271/sjweh.948

Laidra K, Allik J, Harro M, Merenäkk L and Harro J (2006) Agreement among adolescents, parents, and teachers on adolescent personality. *Assessment* **13**, 187–196. doi: 10.1177/1073191106287125

Larsen RJ (1984) Theory and measurement of affect intensity as an individual difference characteristic. *Dissertation Abstracts International* 85, 2297B

Lehto K, Mäestu J, Kiive E, Veidebaum T, Harro J (2016) BDNF Val66Met genotype and neuroticism predict life stress: A longitudinal study from childhood to adulthood. *European neuropsychopharmacology*, *26*(3), 562–569. doi: 10.1016/j.euroneuro.2015.12.029

Logan GD, Schachar RJ and Tannock R (1997) Impulsivity and inhibitory control. *Psychological Science* **8**, 60-64. doi: 10.1111/j.1467-9280.1997.tb00545.x

Lynn R, Allik J, Pullmann H and Laidra K (2002) A study of intelligence in Estonia. *Psychological Reports* **91**, 1022–1026. doi: 10.2466/pr0.2002.91.3.1022

Merenäkk L, Mäestu J, Nordquist N, Parik J, Oreland L, Loit HM and Harro J (2011) Effects of the serotonin transporter (5-HTTLPR) and α_2A_-adrenoceptor (C-1291G) genotypes on substance use in children and adolescents: a longitudinal study. *Psychopharmacology (Berl)* **215**, 13-22. doi: 10.1007/s00213-010-2109-z

Montgomery SA and Åsberg M (1979) A new depression scale designed to be sensitive to change.  *British Journal of Psychiatry* **134**, 382–389. doi: 10.1192/bjp.134.4.382

Mõttus R, Pullmann H and Allik J (2006) Toward more readable Big Five Personality Inventories. *European Journal of Psychological Assessment* **22**, 149–157. doi/10.1027/1015-5759.22.3.149

Paaver M, Eensoo D, Pulver A and Harro J (2006) Adaptive and maladaptive impulsivity, platelet monoamine oxidase (MAO) activity and risk-admitting in different types of risky drivers. *Psychopharmacology (Berl)* **186**, 32-40. doi: 10.1007/s00213-006-0325-3

Paaver M, Kurrikoff T, Nordquist N, Oreland L and Harro J (2008) The effect of 5-HTT gene promoter polymorphism on impulsivity depends on family relations in girls. *Progress in Neuro-Psychopharmacology & Biological Psychiatry* **32**, 1263-1268. doi: 10.1016/j.pnpbp.2008.03.021

Patton JH, Stanford MS and Barratt ES (1995) Factor structure of the Barratt impulsiveness scale. *Journal of Clinical Psychology* **51**, 768–774. https://doi.org/10.1002/1097-4679(199511)51:6<768::aid-jclp2270510607>3.0.co;2-1

Podar I, Hannus A and Allik J (1999) Personality and affectivity characteristics associated with eating disorders: a comparison of eating disordered, weight-preoccupied, and normal samples. *Journal of Personality Assessment* **73**, 133–147. doi: 10.1207/S15327752JPA730109

Pullmann H and Allik J (2000) The Rosenberg Self-Esteem Scale: Its dimensionality, stability and personality correlates in Estonian. *Personality and Individual Differences* **28**, 701–715. doi: 10.1016/S0191-8869(99)00132-4

Raven J, Raven JC and Court JH (1998) Manual for Raven's Progressive Matrices and Vocabulary Scales, Section 1: General Overview. San Antonio, TX: Harcourt Assessment.

Rohner, R. P., & Khaleque, A. (2005). Parental acceptance-rejection questionnaire (PARQ): Test manual. *Handbook for the study of parental acceptance and rejection*, *4*, 43-106

Rosenberg M (1965) Rosenberg self-esteem scale (RSE). *Acceptance and Commitment Therapy*. Measures Package, 61.

Sheehan DV, Lecrubier Y, Sheehan KH, Amorim P, Janavs J, Weiller E, Hergueta T, Baker R and Dunbar GC (1998) The Mini-International Neuropsychiatric Interview (M.I.N.I.): the development and validation of a structured diagnostic psychiatric interview for DSM-IV and ICD-10.  *Journal of Clinical Psychiatry* **59** (Suppl 20), 22–57.

Shlik J, Aluoja A and Kihl E (1999) MINI 5.0.0. Mini rahvusvaheline neuropsühhiaatriline intervjuu DSM – IV. Estonian version of MINI international neuropsychiatric interview.

Spielberger CD, Gorsuch RL, Lushene PR, Vagg PR and Jacobs AG (1983) Manual for the State-Trait Anxiety Inventory. Consulting Psychologists Press, Inc., Palo Alto.

Tulviste T (2013) Socialization values of mothers and fathers: Does the child’s age matter? *Trames* 17(67/62),2. 129-140. DOI: 10.3176/tr.2013.2.02

Tulviste, T., Kiive, E., Akkermann, K., & Harro, J. (2015). Fears in the General Population: More Frequent in Females and Associated With the Serotonin Transporter Promoter Polymorphism and Perceived Relationship With Mothers. *Journal of child neurology*, *30*(11), 1459–1465. https://doi.org/10.1177/0883073815570151
